# Exploring Redox States, Doping and Ordering of Electroactive Star‐Shaped Oligo(aniline)s

**DOI:** 10.1002/chem.201603527

**Published:** 2016-10-10

**Authors:** Benjamin M. Mills, Natalie Fey, Tomasz Marszalek, Wojciech Pisula, Patrice Rannou, Charl F. J. Faul

**Affiliations:** ^1^ School of ChemistryUniversity of BristolBristolBS8 1TSUK; ^2^ Max Planck Institute for Polymer ResearchAckermannweg 1055128MainzGermany; ^3^ Department of Molecular PhysicsFaculty of ChemistryLodz University of TechnologyZeromskiego 11690-924ŁódźPoland; ^4^ Université Grenoble Alpes, INAC-SPrAM38000GrenobleFrance; ^5^ CNRS, INAC-SPrAM38000GrenobleFrance; ^6^ CEA, INAC-SPrAM38000GrenobleFrance

**Keywords:** density functional calculations, oligo(aniline)s, pi-conjugated materials, star-shaped molecules, structure-property relationships

## Abstract

We have prepared a simple star‐shaped oligo(aniline) (**TDPB**) and characterised it in detail by MALDI‐TOF MS, UV/Vis/NIR spectroscopy, time‐dependent DFT, cyclic voltammetry and EPR spectroscopy. **TDPB** is part of an underdeveloped class of π‐conjugated molecules with great potential for organic electronics, display and sensor applications. It is redox active and reacts with acids to form radical cations. Acid‐doped **TDPB** shows behaviour similar to discotic liquid crystals, with X‐ray scattering investigations revealing columnar self‐assembled arrays. The combination of unpaired electrons and supramolecular stacking suggests that star‐shaped oligo(aniline)s like **TDPB** have the potential to form conducting nanowires and organic magnetic materials.

## Introduction

π‐Conjugated materials are being used in an increasingly wide range of organic electronic devices, such as field‐effect transistors,^1^ light‐emitting diodes,^2^ solar cells^3^ and gas sensors,^4^ to name but a few. One of the best‐known and most versatile systems is poly(aniline) (PANI), owing to its unique acid‐induced conductivity, its wide range of oxidation states with different colours^5^ and its reactive nitrogen atoms that can bind small molecules.^6^ Oligo(aniline)s are well‐defined oligomers of PANI^7^ that overcome some of the factors limiting PANI's conductivity, such as polydispersity^8^ and microphase segregation.^9^ They have a strong propensity to self‐assemble, which can be tuned through a variety of routes. For example, carefully chosen acid surfactant dopants^10^ were shown to improve their in‐ and out‐of‐plane order, attractive for thin film applications.^11^ Modifying the periphery of oligo(aniline)s provides a further route to tune self‐assembly: attachment of alkyl tails promotes the formation of ordered conducting thin films,^12^ while the addition of a surfactant head‐group to tetra(aniline) causes it to self‐assemble into conducting nanowires in water.^13^ Optoelectronic properties can also be tuned using an oligomer approach: variation of the aromatic ring system at the centre of the molecule leads to systematic changes of optoelectronic properties.^14^ Finally, the presence of external templates such as graphene has been used to direct the crystallization of oligo(aniline)s vertically, orienting the molecules into stacks and increasing the conductivity by an order of magnitude.^15^ This approach has recently been extended to other π‐conjugated molecules to form optical microcavities for use in nanolasers.^16^

One promising and somewhat underdeveloped approach to tuning self‐assembly and optoelectronic properties is through exploring the use of non‐linear π‐conjugated structures,^17^ such as star‐shaped architectures (i.e., molecules with three or more linear “arms” connected to a central “core”).^18^ Star‐shaped π‐conjugated oligomers have already received some attention,^19^ particularly those based on thiophenes,^20^ fluorenes^21^ and triarylamines.^22^ Compared with their linear counterparts, they show marked differences in their absorption spectra, solubility, self‐assembly and thermal stability. Reviews have highlighted the use of star‐shaped oligomers as active layers in organic thin‐film transistors^23^ (OTFTs) and solution‐processable organic photovoltaics (OPVs).^24^ Star‐shaped oligomers also share structural features with discotic mesogens that can allow them to form ordered liquid‐crystalline phases,^25^ of great interest for organic electronics.^26^ However, there are very few reports of star‐shaped oligo(aniline)s,^27^ leaving many unanswered questions about the fundamental behaviour of this class of π‐conjugated molecules. In light of the potentially desirable properties and applications of star‐shaped oligo(aniline)s, and the lack of attention given to them in the literature, we present here the results of a detailed study of the redox behaviour, acid doping, optoelectronic properties and self‐assembly of a simple star‐shaped oligo(aniline) derivative. From these investigations we aim to develop tools to understand, design and exploit this class of materials for future applications.

## Results and Discussion

**Scheme 1:**
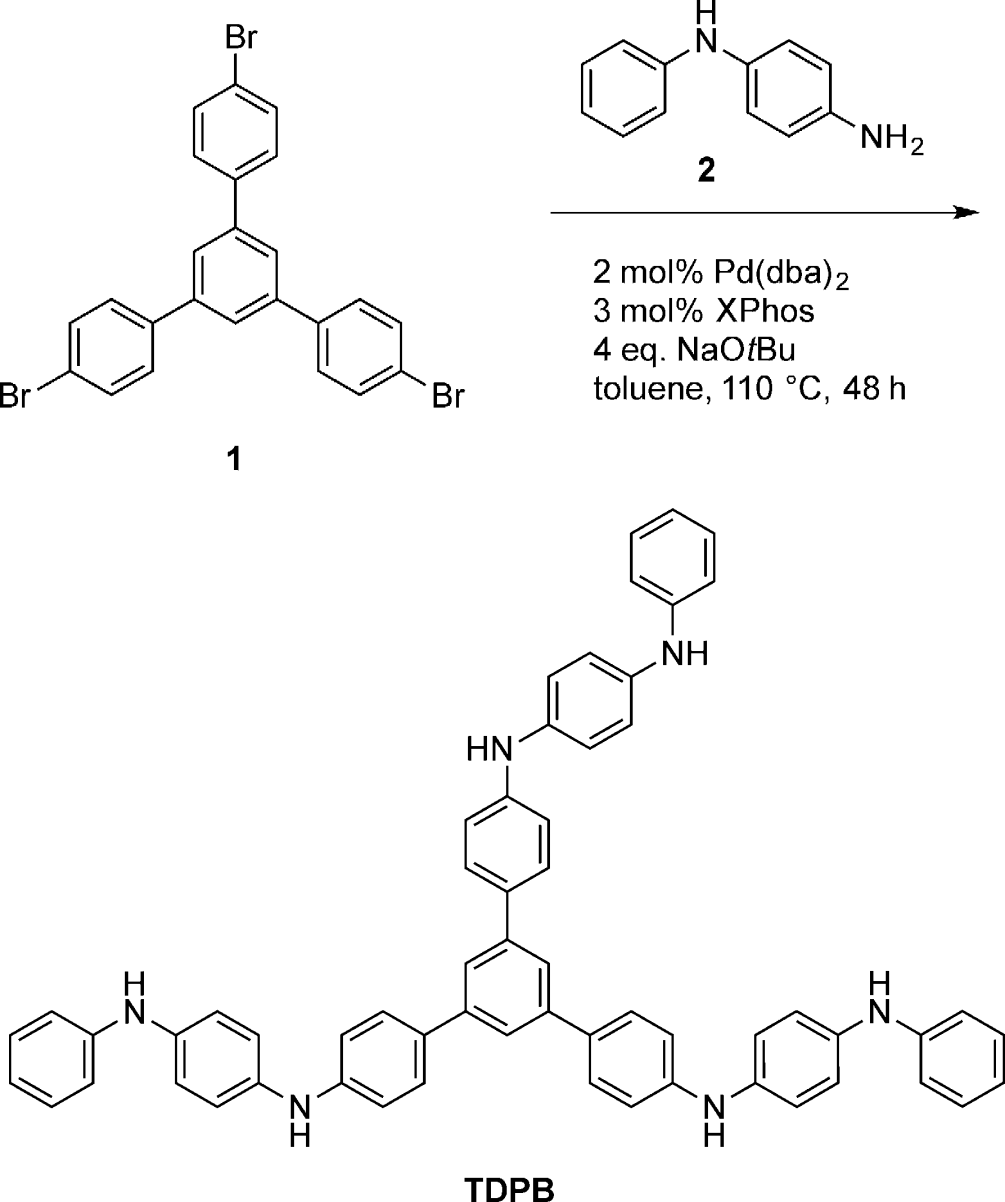
Synthetic route to the star‐shaped oligo(aniline) **TDPB** in its fully reduced state.

By coupling a star‐shaped tribromide **1** with dianiline **2** (Scheme 1) using the Buchwald–Hartwig reaction,^14^ a simple star‐shaped oligo(aniline) was obtained in its fully reduced state in high yield (see the Supporting Information for all experimental and analytical details). The product, 1,3,5‐tris(4‐dianilinophenyl)benzene, is abbreviated hereafter to **TDPB**.

**Scheme 2:**
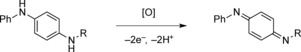
Structural changes upon full oxidation of dianiline arms.

**Figure 1:**
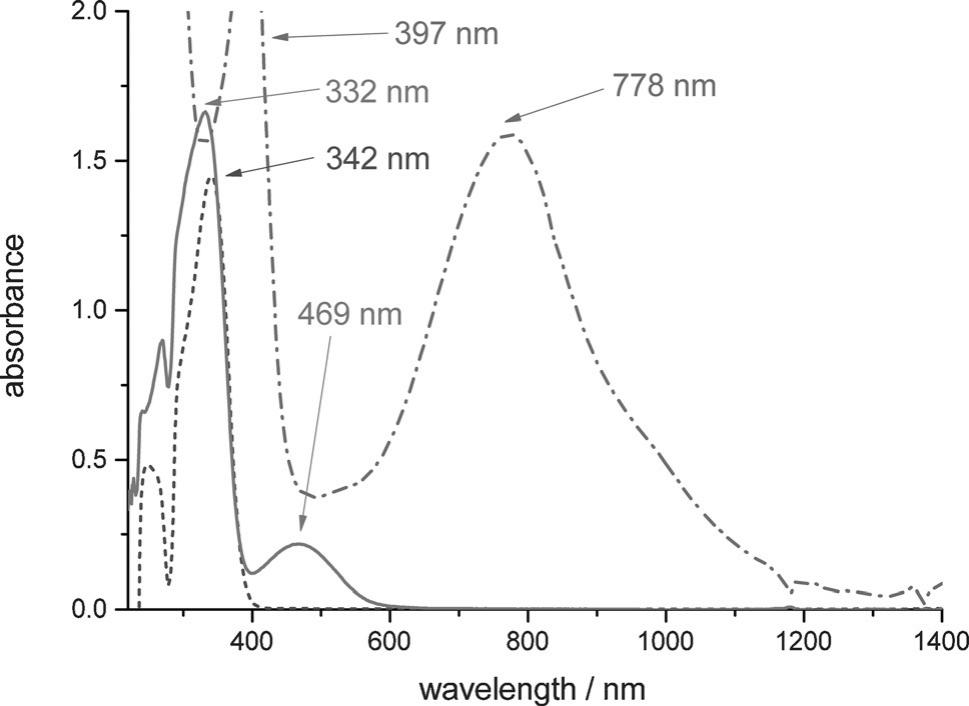
Solution‐state UV/Vis/NIR spectra of **TDPB** in the reduced state (dashed line), after oxidation by air (solid line), and doped (0.15 mm) with (±)‐camphor‐10‐sulfonic acid (1.00 mm) in THF (dash‐dot line).

**TDPB** is redox active: as a solid and in solution it is gradually oxidised in air, changing in colour from pale brown to red‐orange. MALDI‐TOF mass spectrometry revealed that a mixture of redox states is always present. Species with zero, one, two and three arms in the oxidised quinoid form^28^ (Scheme 2) were observed (see Supporting Information Figure S4–S9). The spectra indicate a complex mixture of ionised and protonated species that arise from electron and proton transfers between matrix and analyte molecules. See Supporting Information Table S1 for a detailed listing of these species and their masses, depending on redox state. Strong oxidants such as Ag_2_O ensured a distribution in favour of the more highly oxidised states, but also led to some decomposition. Oxidised samples of **TDPB** are easily identified by their UV/Vis/NIR spectra (Figure 1), which show a characteristic peak at 469 nm, and no further absorption at higher wavelengths.

In order to characterise this seemingly random mixture of redox states, we turned to simulations to provide further insight. Time‐dependent density functional theory (TD‐DFT) simulations are very useful for identifying electronic states of oligo(aniline)s in solution that are difficult to isolate or characterise in full.^13^, ^14^

**Figure 2:**
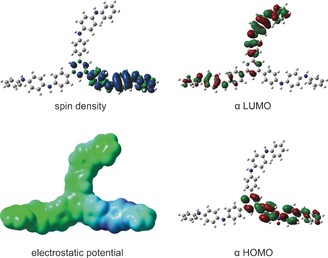
Illustrative surfaces showing some of the calculated molecular properties of the radical cationic state of **TDPB**.

We used a two‐step process to model **TDPB**: geometry minimisation, followed by simulation of the UV/Vis/NIR spectrum. We based our initial structures on the isomers and conformations of other oligo(aniline)s determined by X‐ray diffraction,^29^ NMR spectroscopy,^30^ and STM,^31^ then minimised the geometries in Gaussian^32^ with a widely used DFT method: the B3LYP functional,^33^ the 6‐31G* basis set,^34^ and a polarisable continuum model^35^ (PCM) to account for solvation. The output of this first modelling step can be examined in a variety of ways,^36^ some of which are illustrated in Figure 2 for the radical cation of **TDPB**. In the second step, we used the CAM‐B3LYP functional^37^ to simulate UV/Vis/NIR spectra, since it accounts for charge‐transfer excitations and polarizability in extended π‐conjugated systems better than B3LYP does, and therefore provides more accurate simulated spectra.^38^

TD‐DFT simulations yielded similar UV/Vis/NIR absorption maxima (in the range 445–450 nm) for all the possible oxidised states of **TDPB**, that is, with one, two or three arms oxidised. These results imply that any mixture of these states should have practically indistinguishable UV/Vis/NIR spectra. The spectral similarity of the oxidised forms of **TDPB** shows the arms have little electronic influence on one another, and act as isolated dianiline species. These observations are consistent with absorption spectra of other π‐conjugated molecules with *meta* substitution at a benzene ring.^40^ Full details of the DFT calculations are included in the Supporting Information, including comparisons with the simple dimeric linear analogue, **DPPD**, which shows very similar experimental and simulated UV/Vis/NIR spectra of the reduced and oxidised states (Supporting Information Figure S39–S44 and Table S15).

**Scheme 3:**
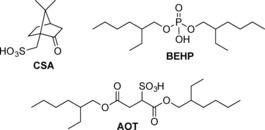
Structures of organic Brønsted acids used to dope **TDPB**.

Oxidised solid samples of PANI and oligo(aniline)s typically form a conducting state (emeraldine salt or ES) upon addition of Brønsted or Lewis acids. The semiconducting to conducting transition is marked by a colour change from blue to green and accompanied, in PANI, by an increase in DC conductivity values of approximately 10 to 13 orders of magnitude.^41^ Addition of Brønsted acids to the oxidised form of **TDPB** in THF or ethanol solutions caused a comparable colour change from red to green. When a strong aqueous mineral acid such as HCl, HBr or H_2_SO_4_ was used, a precipitate formed within a few seconds, leaving a colourless supernatant after a few minutes (see Supporting Information Figure S1). In contrast, protonic doping with organic Brønsted acids (Scheme 3) led to stable, clear solutions that did not form a precipitate. Three protonic dopants, widely used in previous studies of PANI and oligo(aniline)s, were also compared here: (±)‐camphor‐10‐sulfonic acid (**CSA**), bis(2‐ethylhexyl)sulfosuccinic acid (**AOT**) and bis(2‐ethylhexyl) hydrogenphosphate (**BEHP**). All three reacted with **TDPB** in THF to form solutions showing a UV/Vis/NIR absorption band with a maximum at approximately 775 nm. See Figure 1 for a representative spectrum of the **CSA**‐doped species (and Supporting Information, Figure S16 and S17 for **AOT** and **BEHP** data, respectively).

**Figure 3:**
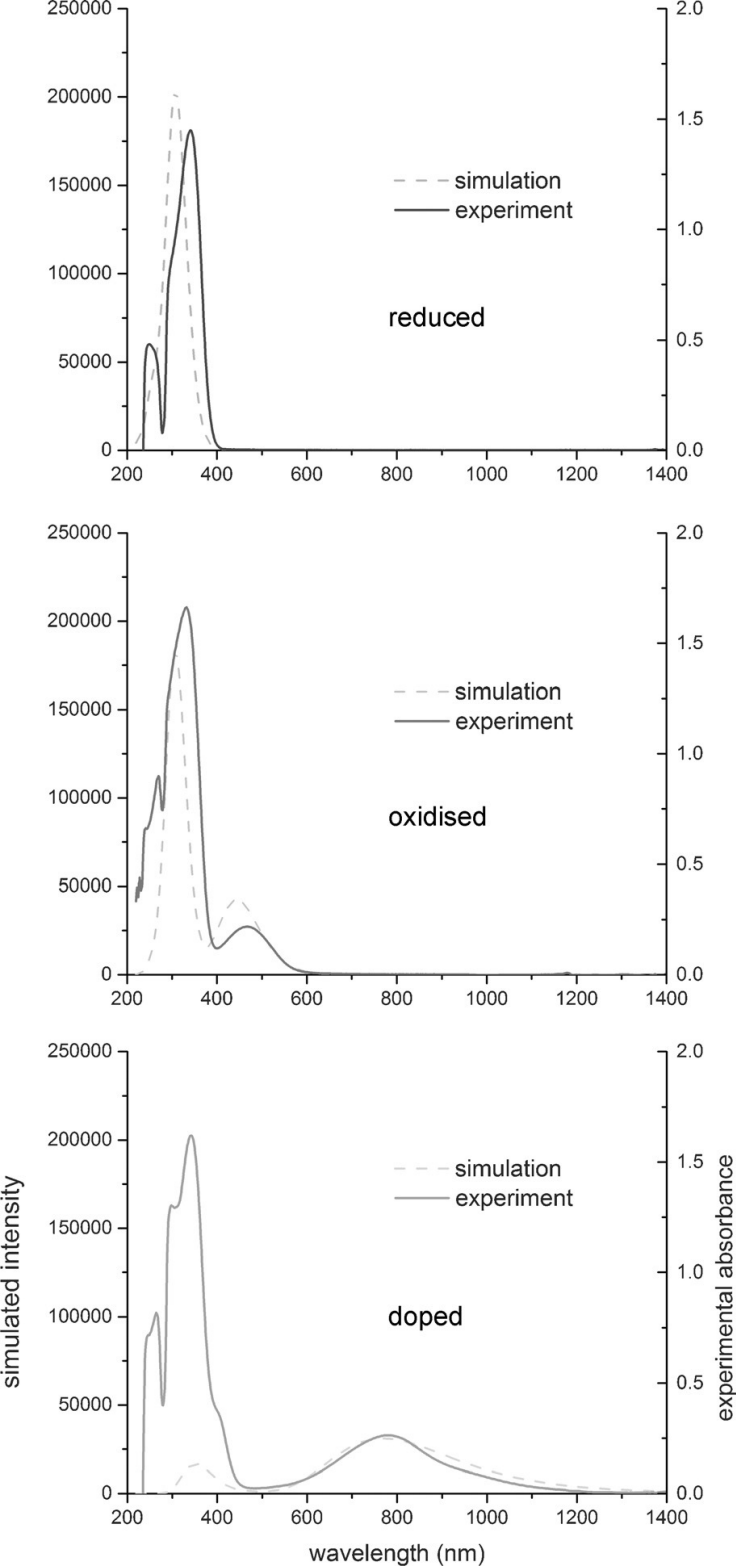
Comparison of simulated^39^ (CAM‐B3LYP, dashed lines) and experimental spectra (THF, solid lines) for the reduced, oxidised and doped states of **TDPB**. For simplicity, counterions were not modelled.

TD‐DFT simulations assisted in identifying the species responsible for the experimentally observed UV/Vis/NIR absorption (Figure 3). The radical cation of **TDPB** is a good match for the observed spectra, with a calculated UV/Vis/NIR maximum of 772 nm, compared to the experimental maxima of approximately 775 nm. Phenyl‐capped dianiline (**DPPD**) forms a similar species with a similar absorption spectrum in acidic conditions.^28^ The singlet dication can be ruled out with a very high level of confidence as the major doped state of **TDPB**, as its simulated UV/Vis/NIR maximum of 923 nm is 150 nm higher than the experimental value. Other possibilities that cannot be ruled out include a triplet dicationic diradical, and doublet and quartet tricationic triradical species^42^ (calculated *λ*_max_ values of 755, 750 and 751 nm, respectively; see Supporting Information for further details). These results clearly show that **TDPB** forms species containing unpaired electrons when oxidised samples are exposed to acids.

**Figure 4:**
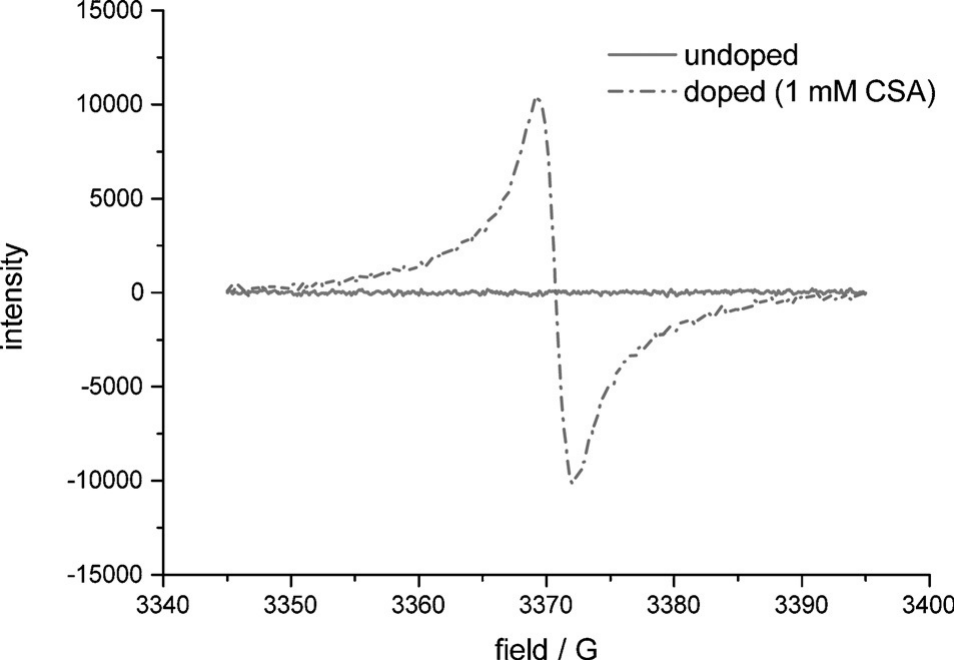
EPR spectra of undoped (no **CSA** added) and doped (1.00 mm **CSA**) solutions of **TDPB** (0.15 mm) in THF.

Electron paramagnetic resonance (EPR) spectroscopy showed no response for acid‐free oxidised solutions, as expected for a closed‐shell, diamagnetic species (Figure 4, solid line). However, the presence of radicals in acid‐doped solutions of **TDPB** was confirmed: after **CSA** was added, a signal appeared at 3370 G, indicating the formation of an open‐shell, paramagnetic doped species (Figure 4, dotted line).

**Figure 5:**
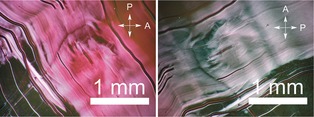
Polarized light micrographs of birefringent, sheared, soft films of **TDPB** doped with **AOT**, drop cast from an octan‐1‐ol solution.

**TDPB**’s triphenylbenzene core gives it the potential to act as a discotic mesogen.^43^ Although **TDPB** is not able to form mesophases on its own due to the lack of soft and flexible peripheral alkyl groups, such groups can be introduced through doping. It has been shown that oligo(aniline)s doped with acid surfactants can form supramolecular thermotropic liquid‐crystalline phases,^10^ whilst lyotropic PANI liquid crystals were obtained when doped by **CSA** in a *m*‐cresol solution.^44^ Following a similar strategy, solutions of oligomers doped with **CSA** or **AOT** were drop‐cast onto hydrophobized glass slides and investigated by polarized light microscopy (PLM) to reveal any birefringent textures and thus anisotropic organization. When low‐boiling solvents such as THF, ethanol and propan‐1‐ol were used, the solvents evaporated quickly to leave very viscous films that could not be sheared by hand. When using octan‐1‐ol (a higher boiling point solvent used in previous tetra(aniline)‐based studies^45^) slow evaporation led to the formation of soft phases that exhibited strong birefringence when sheared (Figure 5). The sheared films did not undergo any visible phase transitions upon heating until their decomposition around 180 °C.

**Figure 6:**
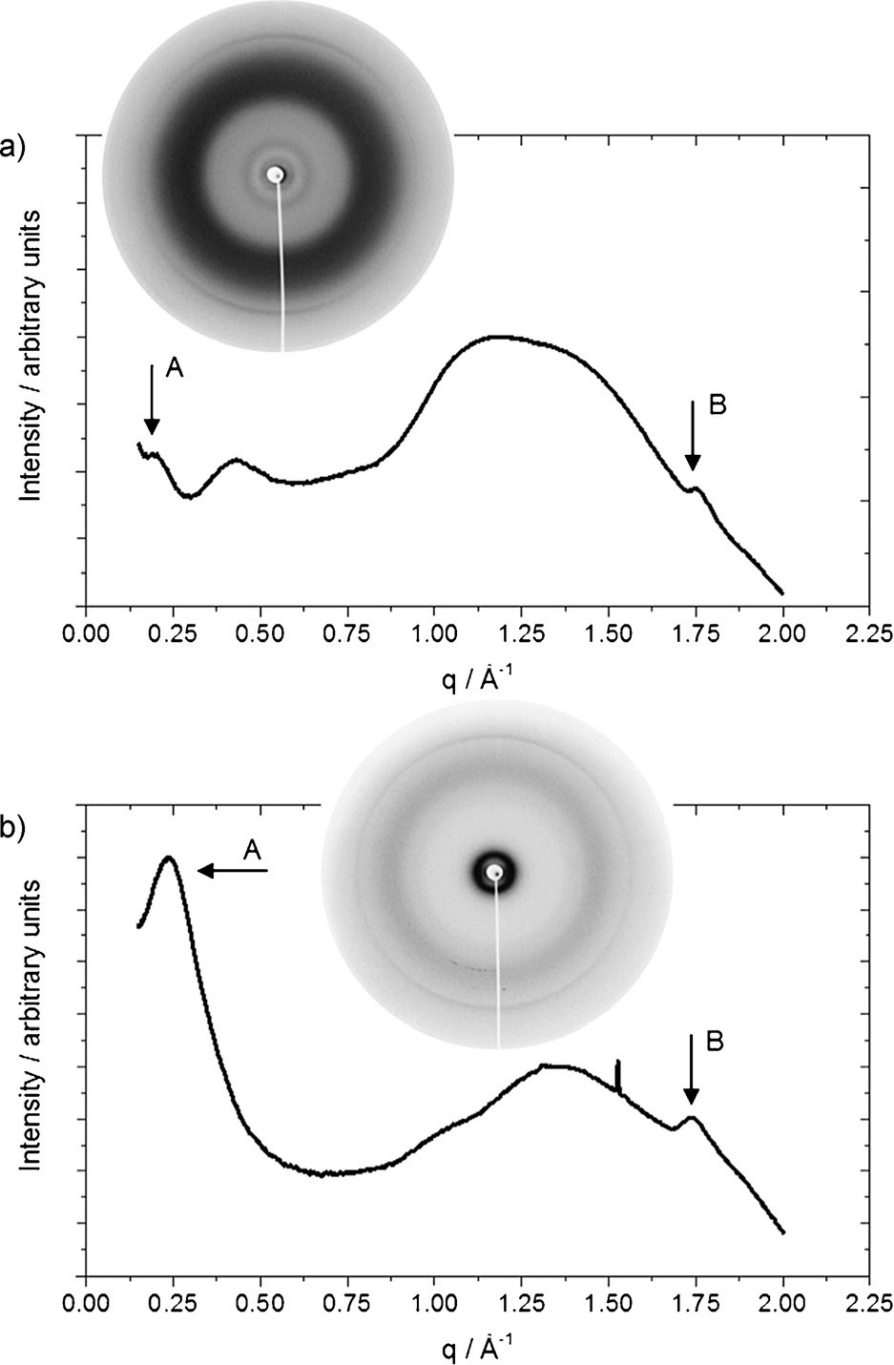
Line profiles (intensity vs. scattering vector (q)) from 2D WAXS patterns of **TDPB** doped with a) **CSA** and b) **AOT** (A: interstack and B: π‐stacking peaks); insets: corresponding 2D WAXS patterns of extruded filaments.

To determine the exact nature of supramolecular ordering leading to the observed birefringence, we performed two‐dimensional wide‐angle X‐ray scattering (2D WAXS) on extruded filaments of the doped **TDPB** materials. In short, solutions of **TDPB** in THF were doped with **CSA** or **AOT** and the solvent allowed to evaporate. Samples were then prepared according to a previously published method,^46^ and the solid residue heated to 70 °C. A thin filament of the heated material was extruded (a process similar to shearing of soft films), placed in an X‐ray beam, and the resulting X‐ray scattering pattern recorded with a 2D area detector (insets in Figure 6).

A number of important features are notable in the 2D patterns: 1) there is very little orientational dependence in the scattering patterns; 2) a relatively intense amorphous halo is observed in both cases, consistent with disordered acid dopant molecules surrounding more ordered aggregates of **TDPB** molecules; 3) two prominent but isotropic reflections are obvious in the patterns for **TDPB** doped with **CSA** and **AOT**. The features in the small‐angle region (A) are attributed to stacks that are formed by the **TDPB** molecules, while the wide‐angle reflection (B) is attributed to the typical intermolecular π‐stacking distance of 0.36 nm. The scattering patterns are clearly influenced by the nature and steric bulk of the dopant, as reflected by the inter‐stack distance (Table 1). The π‐stacking distance is independent of the dopant. Taken together, the X‐ray data suggest a loosely ordered material containing stacks of **TDPB** molecules (stacked with the typical π‐stacking distance of 0.36 nm), separated by regions of disordered acid dopants.

**Table 1:** Key data from 2D WAXS experiments.

Dopant	Interstack distance [nm]	π–π stacking distance [nm]
**CSA**	3.25	0.36
**AOT**	2.91	0.36

Further experiments with tailor‐made pro‐mesogenic protonating agents and varying doping ratios are envisaged to gain further insight into this new class of conducting star‐shaped oligo(aniline)s and any liquid‐crystalline phases formed. In addition, a more rigid core is likely to induce stronger interstack ordering, which may lead to the formation of nanowires. Such nanostructures have been extensively developed for triarylamines22b–22e and observed recently in other oligo(aniline)‐based systems.^13^

## Conclusion

In conclusion, the effect of chemical structure on spectra, spin and supramolecular ordering of the star‐shaped oligo(aniline) derivative **TDPB** has been determined. TD‐DFT simulation of the UV/Vis/NIR spectra of all the oxidation states and doped forms of **TDPB** shows that computational chemistry can be used to characterise and distinguish π‐conjugated materials with complex behaviour. The length of the arms and the π‐conjugation pattern at the core of **TDPB** causes it to form radical cationic species in acidic conditions. The ability to create molecular systems with unpaired electrons is important for applications that rely on electrical conductivity or magnetism. When doped, **TDPB** shows a tendency to self‐assemble into weakly ordered supramolecular columnar aggregates. In future, through further design and structural modifications of this motif (such as planarization of the core of the star‐shaped oligo(aniline) or by using pro‐mesogenic protonating agents), novel radical‐containing supramolecular nanostructures can be created. This work thus provides opportunities for focused future investigations aimed at further developing the full potential of semiconducting and conducting star‐shaped π‐conjugated molecules, and exploring applications, for example, in sensing and generation of reactive oxygen species.

## Experimental Section

*N*‐Phenyl‐1,4‐phenylenediamine (200 mg, 1.09 mmol, 3.1 equiv), 1,3,5‐tris(4‐bromophenyl)benzene (190 mg, 0.35 mmol, 1 equiv), bis(dibenzylideneacetone)palladium(0) (4.0 mg, 7.0 μmol, 2 mol %), XPhos (5.0 mg, 11 μmol, 3 mol %), and sodium *tert*‐butoxide (135 mg, 1.4 mmol, 4 equiv) were added to a round‐bottom flask under nitrogen. Anhydrous toluene (40 mL) was added and the reaction mixture was heated to 110 °C for 2 days. The reaction mixture was then allowed to cool to room temperature, and the solvent was removed under reduced pressure. The residue was dissolved in a minimal volume of THF (ca. 5 mL), phenylhydrazine (120 mg, 3.2 equiv) was added and the mixture was stirred under nitrogen at room temperature for 30 min. Hexane was added (ca. 50 mL) until a pale‐brown‐coloured opaque suspension formed. The suspension was allowed to settle for 1 h, and was then filtered. The precipitate was washed by stirring in water for 1 h, then filtered and freeze dried to afford the product (271 mg, 91 %) as a light brown powder. ^1^H NMR (500 MHz, [D_6_]DMSO): *δ*=8.08 (s, 3 H), 7.91 (s, 3 H), 7.66 (d, *J*=8.6 Hz, 6 H), 7.64 (s, 3 H), 7.18 (dd, *J*=8.5, 7.3 Hz, 6 H), 7.08 (m, 18 H), 6.98 (d, *J*=7.3 Hz, 6 H), 6.73 ppm (t, *J*=7.3 Hz, 3 H); ^13^C NMR (125 MHz, [D_6_]DMSO): *δ*=144.77, 144.58, 141.35, 136.74, 136.05, 130.34, 129.09, 127.69, 121.24, 120.04, 119.58, 118.41, 116.63, 115.17 ppm; IR (neat) 3385, 3025, 1597, 1508, 1494, 1296, 1183, 818, 746, 693, 614 cm^−1^; HRMS‐MALDI *m*/*z* calcd for C_60_H_48_N_6_: 852.3940; found: 852.3942.

## Supporting information

As a service to our authors and readers, this journal provides supporting information supplied by the authors. Such materials are peer reviewed and may be re‐organized for online delivery, but are not copy‐edited or typeset. Technical support issues arising from supporting information (other than missing files) should be addressed to the authors.

Supplementary
