# Supplementary material for: Exploring Redox States, Doping and Ordering of Electroactive Star‐Shaped Oligo(aniline)s
Source: Chemistry. 2016 Oct 10;22(47):16950–6. doi: 10.1002/chem.201603527 (PMC5129507; doi:10.1002/chem.201603527)
Supplement: Supplementary file 1 — Supplementary [file CHEM-22-16950-s001.pdf]

CHEMISTRY
A European Journal

Supporting Information

Exploring Redox States, Doping and Ordering of Electroactive Star-Shaped Oligo(aniline)s

Benjamin M. Mills,[a] Natalie Fey,[a] Tomasz Marszalek,[b] Wojciech Pisula,[b, c] Patrice Rannou,[d, e, f] and Charl F. J. Faul*[a]

chem_201603527_sm_miscellaneous_information.pdf

Electronic supporting information (ESI)

Table of Contents

1. Structures **2**
2. Experimental: **3**
Synthesis of TDPB 3
Mass spectrometry 3
NMR spectroscopy 3
FTIR spectroscopy 3
UV-vis-NIR spectroscopy 3
Cyclic voltammetry 3
DFT calculations 3
EPR spectroscopy 4
Variable-temperature polarized light microscopy 4
2DWAXS experiments 4
Doping experiments 4
3. NMR spectroscopy **5**
^{1}H NMR spectrum 5
^{13}C NMR spectrum 5
4. Mass spectrometry **6**
Reduced state 6
Oxidised states 7
Species present 9
5. UV/vis/NIR spectroscopy **10**
Reduced state 10
Oxidised states 10
Doped states 10
Doped states (continued) 11
6. Cyclic voltammetry **12**
In acidic solution 12
In neutral solution 12
7. Density functional theory (DFT) modelling **13**
Computational approach and methods 13
Reduced state 14
Oxidised state 1 15
Oxidised state 2 18
Oxidised state 3 19
Doped states 21
8. Simulated and experimental spectra of a linear analogue, DPPD **26**
Reduced state 26
Oxidised state 26
Radical cation 27
Summary 27

1. Structures

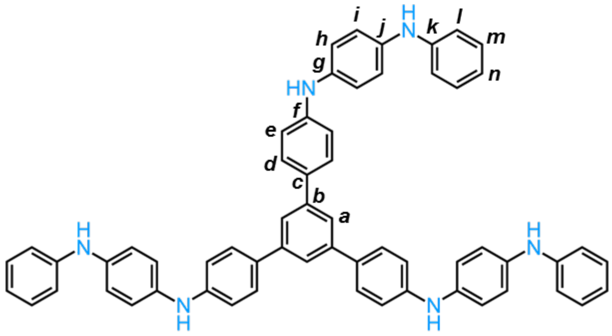

Scheme S1. Structure of **TDPB** with labels for NMR assignment.

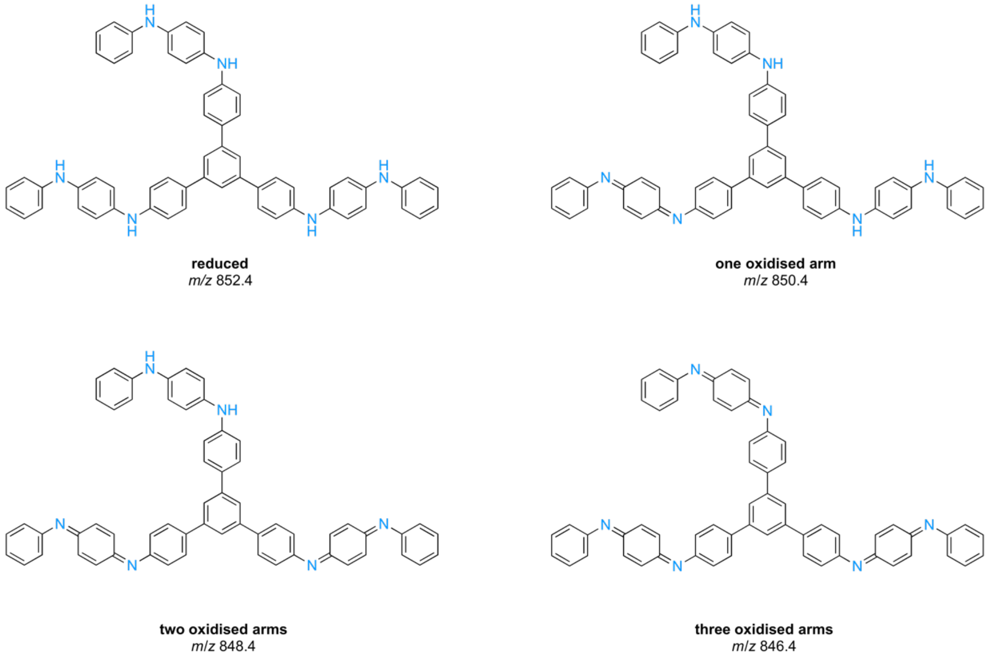

Scheme S2. Structures and *m*/*z* values for the four closed-shell oxidation states of **TDPB**, for mass spectrometry assignment.

2. Experimental:

Synthesis of TDPB

N-Phenyl-1,4-phenylenediamine (200 mg, 1.09 mmol, 3.1 eq.), 1,3,5-tris(4-bromophenyl)benzene (190 mg, 0.35 mmol, 1 eq.), bis(dibenzylideneacetone)palladium(0) (4.0 mg, 7.0 µmol, 2 mol%), XPhos (5.0 mg, 11 µmol, 3 mol%), and sodium *tert*-butoxide (135 mg, 1.4 mmol, 4 eq.) were added to a round-bottom flask under nitrogen. Anhydrous toluene (40 ml) was added and the reaction mixture was heated to 110 °C for 2 days. The reaction mixture was then allowed to cool to room temperature, and the solvent was removed under reduced pressure. The residue was dissolved in a minimal volume of THF (ca. 5 ml), phenylhydrazine (120 mg, 3.2 eq.) was added and the mixture was stirred under nitrogen at room temperature for 30 min. Hexane was added (ca. 50 ml) until a pale-brown coloured opaque suspension formed. The suspension was allowed to settle for 1 h, and was then filtered. The precipitate was washed by stirring in water for 1 h, then filtered and freeze dried to afford the product (271 mg, 91%) as a light brown powder. ^{1}H NMR (500 MHz, DMSO-d_6): δ = 8.08 (s, 3H, inner NH), 7.91 (s, 3H, outer NH), 7.66 (d, $^{3}J_{HH}$ = 8.6 Hz, 6H, **d**), 7.64 (s, 3H, **a**), 7.18 (dd, $^{3}J_{HH}$ = 8.5, 7.3 Hz, 6H, **m**), 7.08 (m, 18H, **e**/**h**/**i**), 6.98 (d, $^{3}J_{HH}$ = 7.3 Hz, 6H, **l**), 6.73 ppm (t, $^{3}J_{HH}$ = 7.3 Hz, 3H, **n**); ^{13}C NMR (125 MHz, DMSO-d_6): δ = 144.77, 144.58, 141.35, 136.74, 136.05, 130.34, 129.09, 127.69, 121.24, 120.04, 119.58, 118.41, 116.63, 115.17 ppm; IR (neat) 3385, 3025, 1597, 1508, 1494, 1296, 1183, 818, 746, 693, 614 cm^{-1}; HRMS-MALDI *m*/*z* calcd for $C_{60}H_{48}N_6$: 852.3940; found: 852.3942. Anal. calcd. for $C_{60}H_{48}N_6$: C, 84.48; H, 5.67; N, 9.85%. Found: C, 83.46; H, 5.56; N, 8.82%.

Mass spectrometry

Samples for MALDI-TOF mass spectra were prepared using of 2,5-dihydroxybenzoic acid (DHB) as a matrix and 2:1 MeCN:water as the solvent. The spectra were run by the University of Bristol's School of Chemistry mass spectrometry service.

NMR spectroscopy

^{1}H and ^{13}C NMR experiments were performed using either a 400 MHz Varian VNMR 400, or a 500 MHz Bruker 500 NMR spectrometer at the University of Bristol's School of Chemistry NMR service.

FTIR spectroscopy

FTIR spectra of neat powder samples were recorded on a PerkinElmer Spectrum Two IR Spectrometer equipped with a UATR Single Reflection Diamond attenuated total reflectance attachment.

UV-vis-NIR spectroscopy

UV-vis-NIR spectra were recorded on a Shimadzu UV2600 spectrophotometer fitted with an ISR-2600Plus integrating sphere attachment.

Cyclic voltammetry

Cyclic voltammograms were recorded with 0.1 M tetrabutylammonium hexafluorophosphate as supporting electrolyte, using an EG&G Princeton Applied Research Model 273 Potentiostat / Galvanostat. The working electrode was a glassy carbon rod (0.07 cm^2), the counter-electrode was a platinum wire, and the reference electrode was a saturated calomel electrode calibrated against an Fc/Fc^+ couple.

DFT calculations

Electronic structure calculations were performed in Gaussian 09 Revision D.01,[31] using the B3LYP method[32] and the 6-31G* basis set. The polarizable continuum model (PCM) was used to account

for solvation.[33] The results of the calculations were visualized in GaussView, Version 5.0.9.[34] Time-dependent DFT calculations were carried out using the CAM-B3LYP method,[35] also with PCM solvation.

EPR spectroscopy

EPR spectra were run by Dr Floriana Tuna at the EPSRC National Service for Electron Paramagnetic Resonance Spectroscopy at the University of Manchester.

Variable-temperature polarized light microscopy

PLM was performed using an Olympus BX-50 optical microscope equipped with an Olympus C-5060 digital camera. Sample temperature was controlled with a Linkam TP92 heater and a Linkam THMS600 heating stage

2DWAXS experiments

2D-WAXS measurements were performed using a custom setup consisting of the Siemens Kristalloflex X-ray source (copper anode X-ray tube, operated at 35 kV/20 mA), Osmic confocal MaxFlux optics, two collimating pinholes (1.0 and 0.5 mm Owis, Germany) and an antiscattering pinhole (0.7 mm – Owis, Germany). The patterns were recorded on a MAR345 image plate detector (Marresearch, Germany). The samples were prepared by filament extrusion using a custom-built mini-extruder and were positioned perpendicular to the incident X-ray beam and vertical to the 2D detector.

Doping experiments

Three 10^{-3} M solutions of oxidised **TDPB** (4.3 mg, 5 µmol) in THF (5 mL) were prepared in 7 ml vials. No dopant was added to the first vial. **CSA** (7.0 mg, 30 µmol, 6 eq.) was added to the second vial. Concentrated HCl(aq) (37%, *ca.* 12 mol L^{-1}, 2.5 µl, 30 µmol, 6 eq.) was added to the third vial. All three vials were left to stand for 10 min before a photograph was taken (Figure S1).

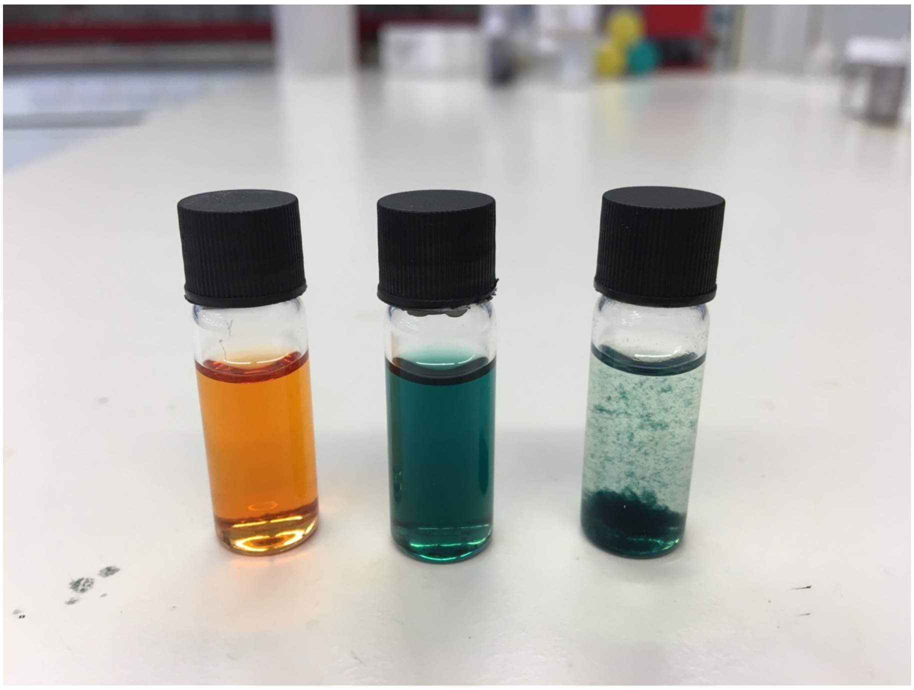

Figure S1. Photograph of THF solutions of oxidised **TDPB** with no dopant added (left), with **CSA** added (centre), and with concentrated aqueous HCl added (right).

3. NMR spectroscopy

^{1}H NMR spectrum

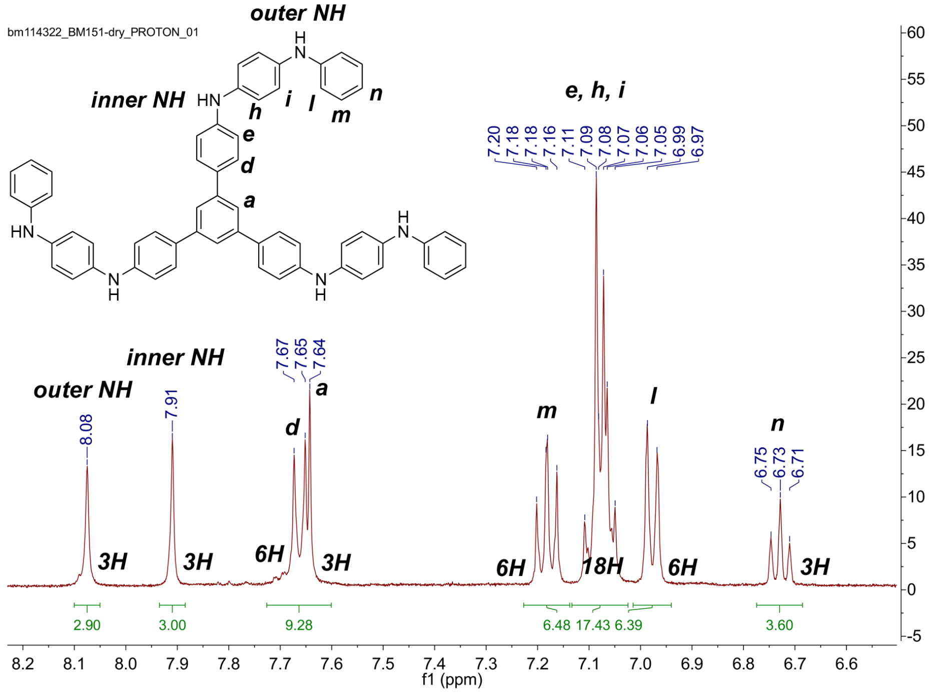

Figure S2. ^{1}H NMR spectrum of **TDPB** in DMSO-d_6

^{13}C NMR spectrum

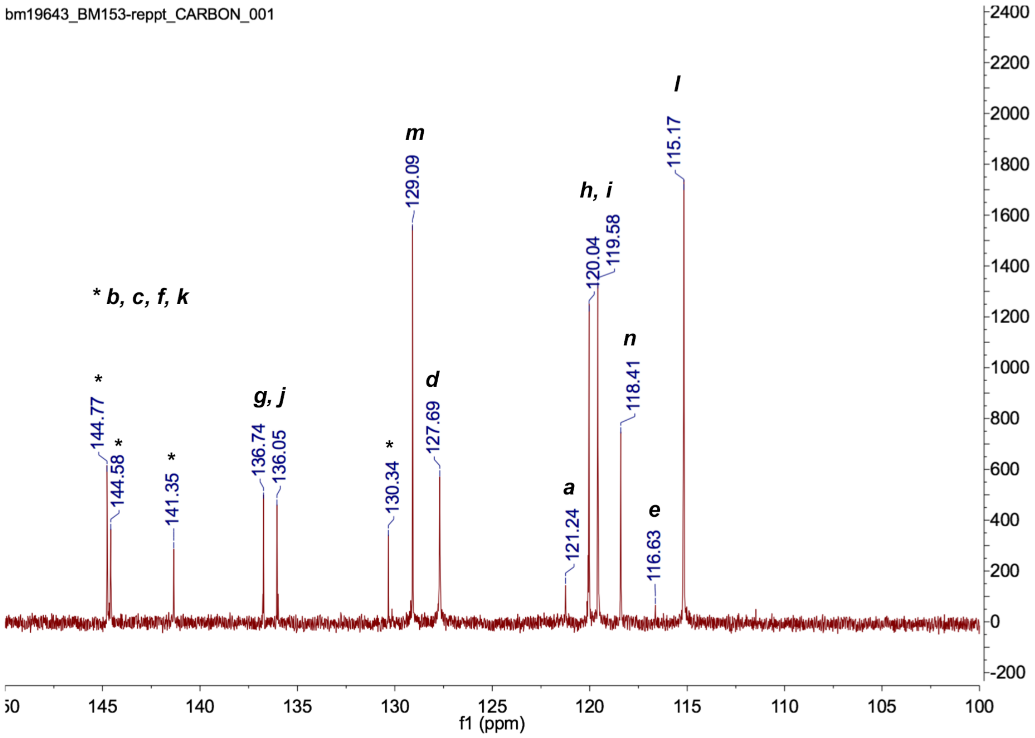

Figure S3. ^{13}C NMR spectrum of **TDPB** in DMSO-d_6

4. Mass spectrometry

Reduced state

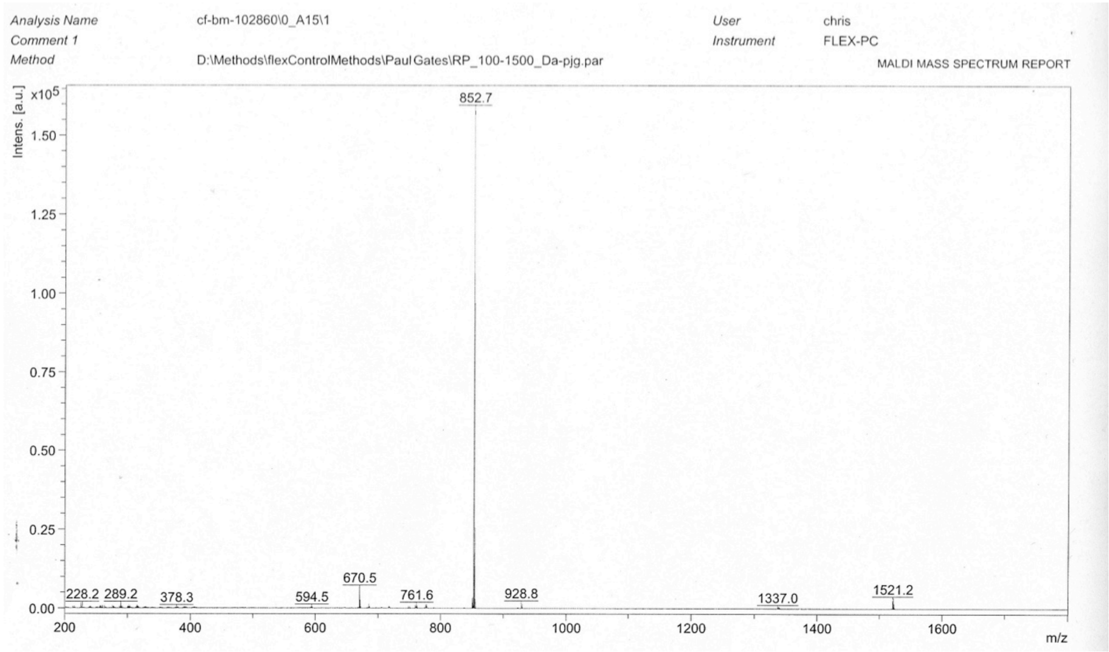

Figure S4. MALDI mass spectrum of **TDPB** in the fully reduced oxidation state.

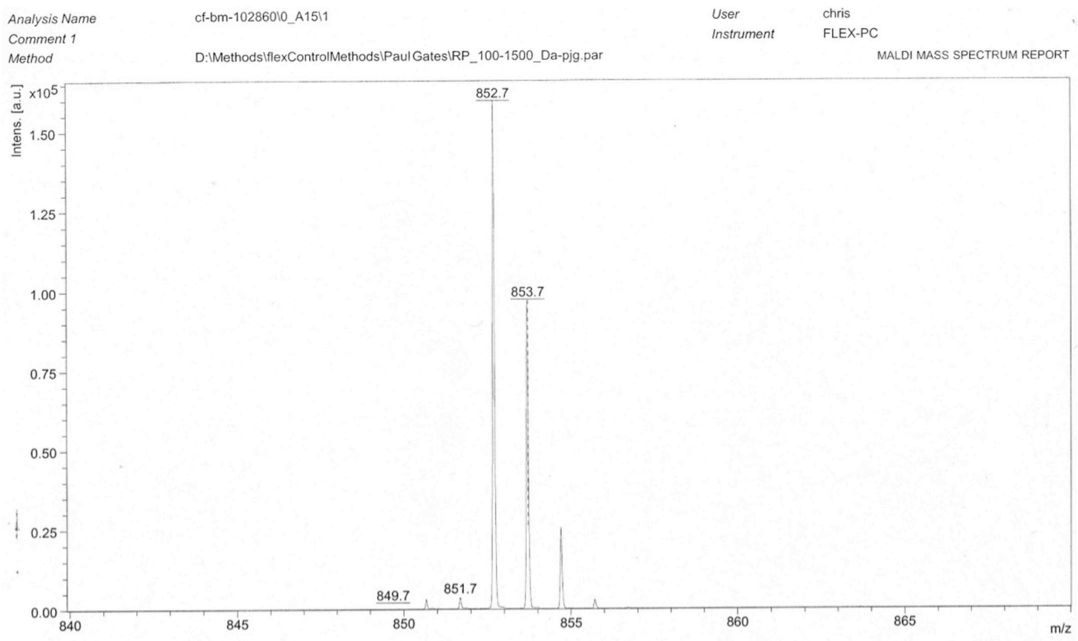

Figure S5. Enlargement of Figure S4 over the *m/z* range 840-870.

Oxidised states

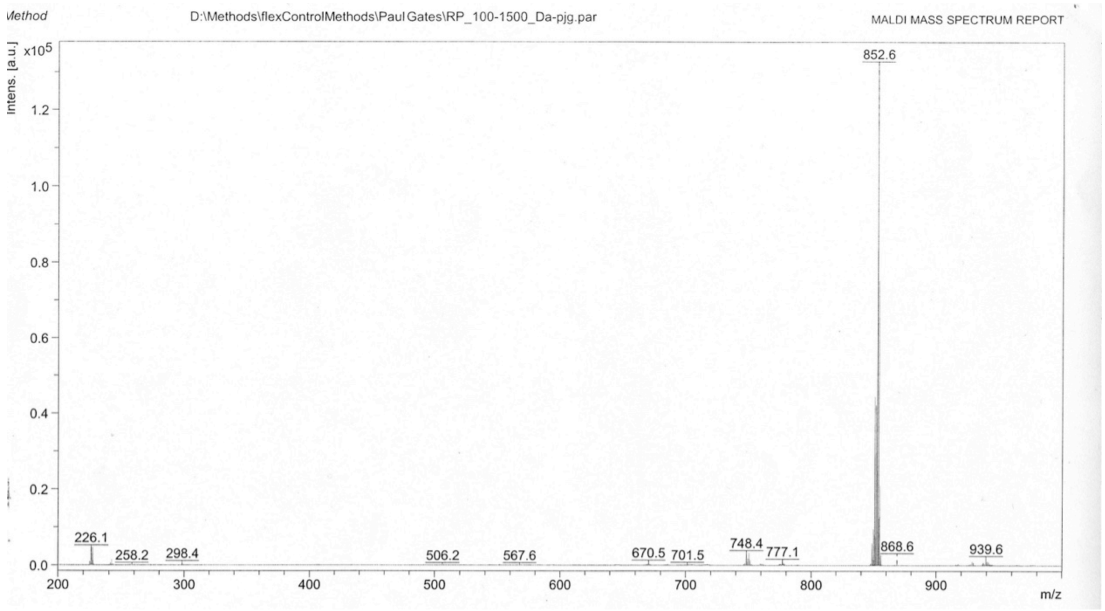

Figure S6. MALDI mass spectrum of **TDPB** after stirring with Ag_2O for 1 h.

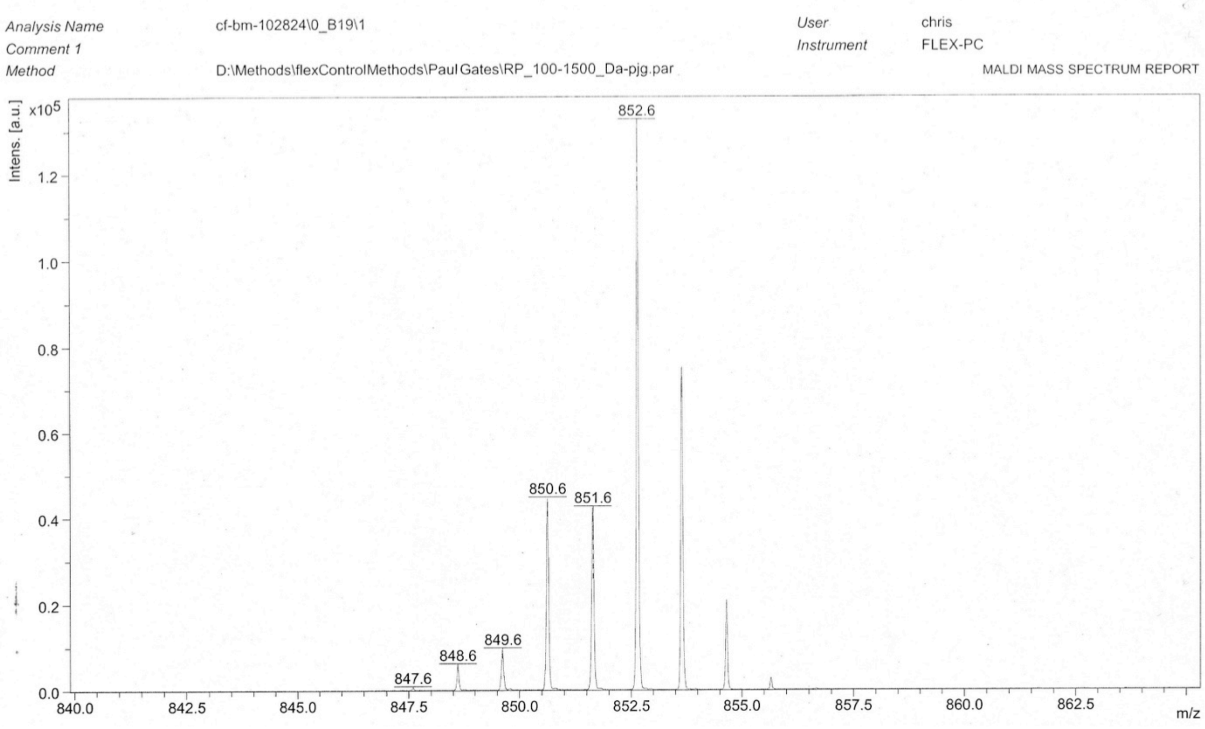

Figure S7. Enlargement of Figure S6 over the *m/z* range 840-870.

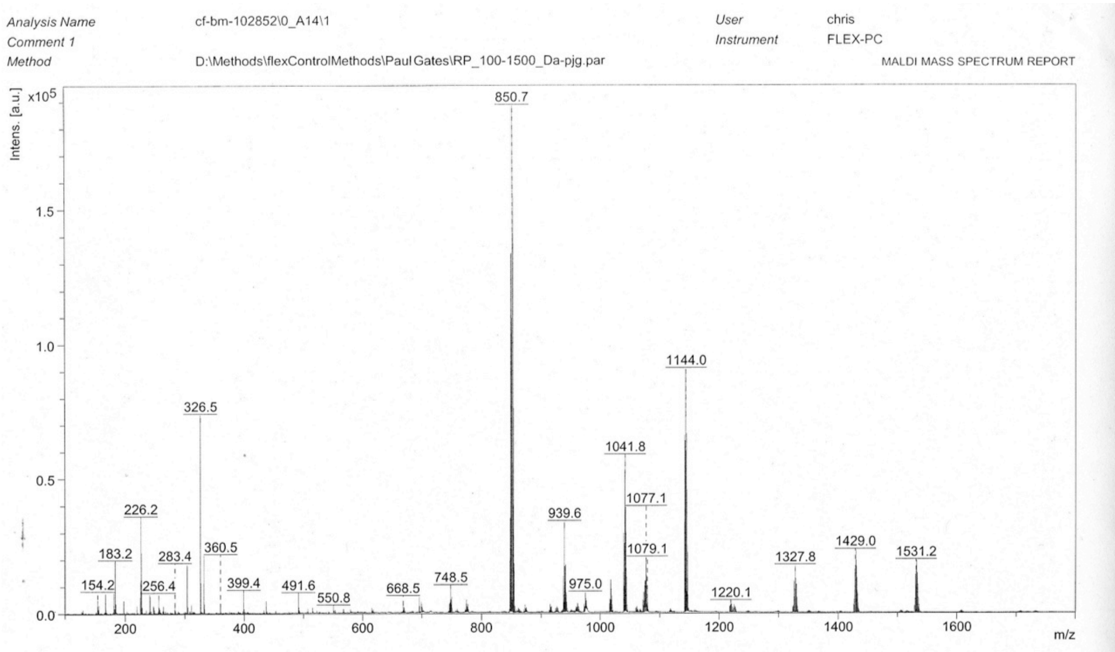

Figure S8. MALDI mass spectrum of **TDPB** after stirring with Ag_2O for 5 d.

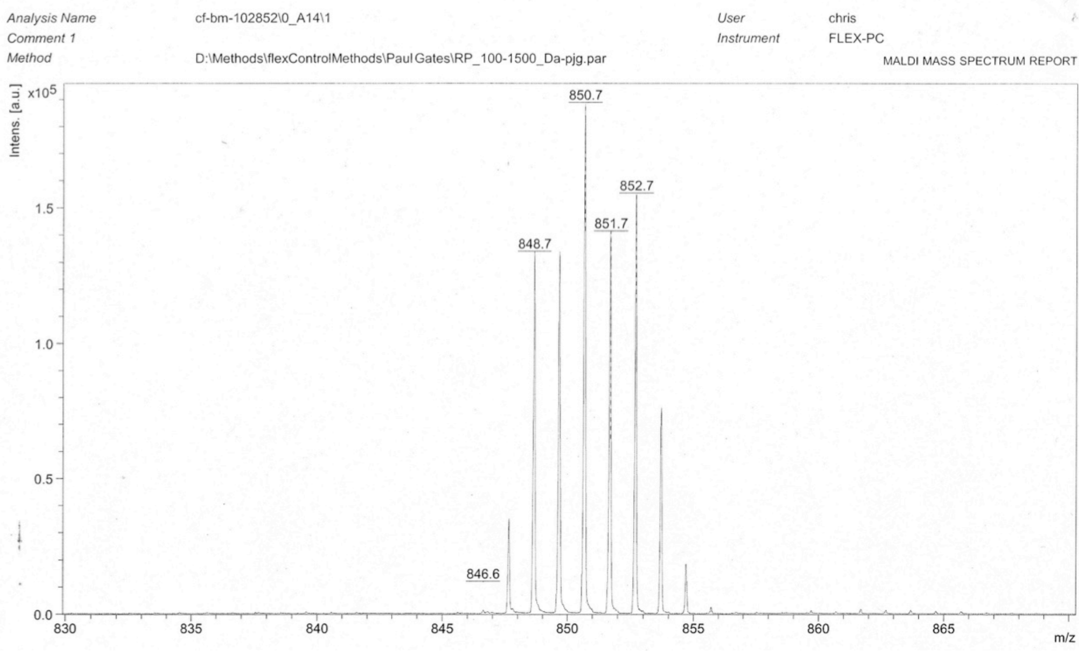

Figure S9. Enlargement of Figure S8 over the *m*/*z* range 830-870.

Species present

Table S1. Composition and *m/z* values for ions observed in the MALDI-TOF mass spectra of **TDPB**.

Structure	Species	Quinoid arms	NH protons	Formula	*m/z*
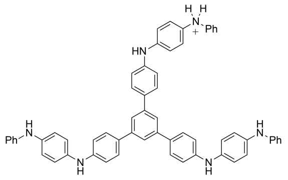	RED+H$^+$	0	7	$C_{60}H_{49}N_6^+$	853.4
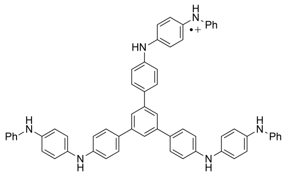	RED	0	6	$C_{60}H_{48}N_6^+$	852.4
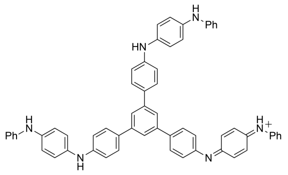	1OX+H$^+$	1	5	$C_{60}H_{47}N_6^+$	851.4
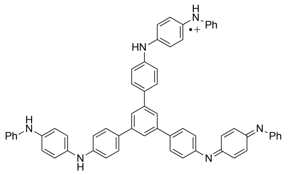	1OX	1	4	$C_{60}H_{46}N_6^+$	850.4
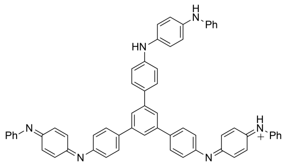	2OX+H$^+$	2	3	$C_{60}H_{45}N_6^+$	849.4
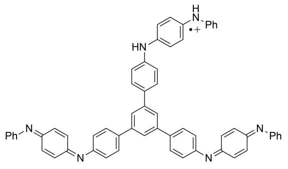	2OX	2	2	$C_{60}H_{44}N_6^+$	848.4
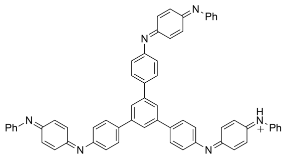	3OX+H$^+$	3	1	$C_{60}H_{43}N_6^+$	847.4
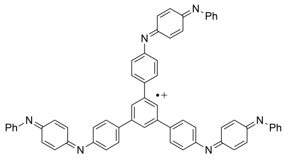	3OX	3	0	$C_{60}H_{42}N_6^+$	846.4

5. UV/vis/NIR spectroscopy

Reduced state

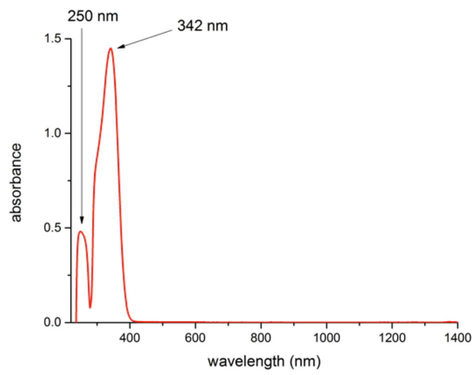

Figure S10. UV/vis/NIR spectrum of **TDPB** in the reduced state, in THF

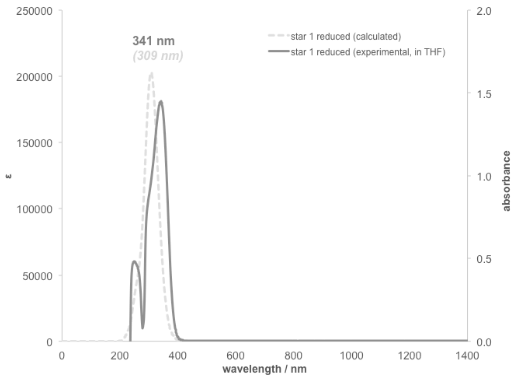

Figure S11. TD-DFT simulated UV/vis/NIR spectrum of **TDPB** in the reduced state, overlaid with experimental

Oxidised states

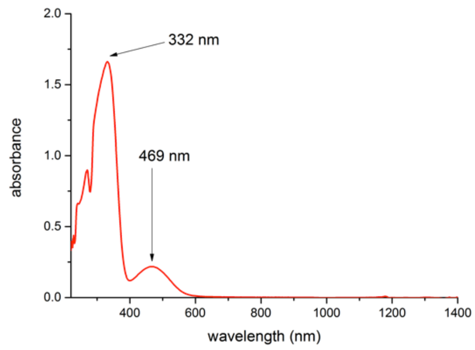

Figure S12. UV/vis/NIR spectrum of **TDPB** after oxidation in air, in THF

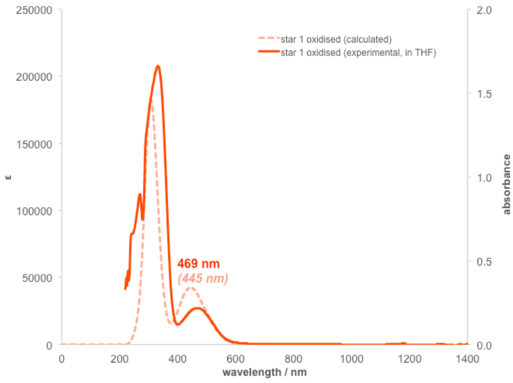

Figure S13. TD-DFT simulated UV/vis/NIR spectrum of **TDPB** with one arm oxidised, overlaid with experimental

Doped states

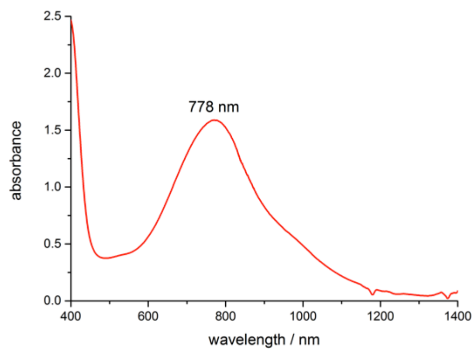

Figure S14. UV/vis/NIR spectrum of oxidised **TDPB** doped with **CSA**, in THF

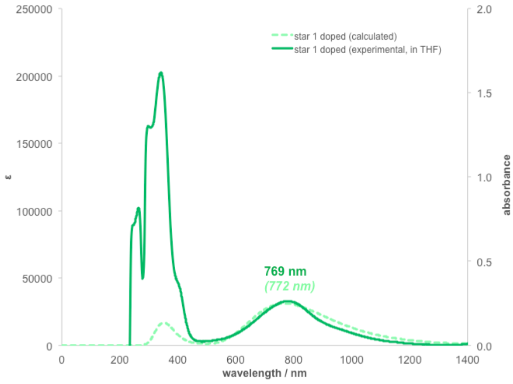

Figure S15. TD-DFT simulated UV/vis/NIR spectrum of the radical cation of **TDPB**, overlaid with experimental

Doped states (continued)

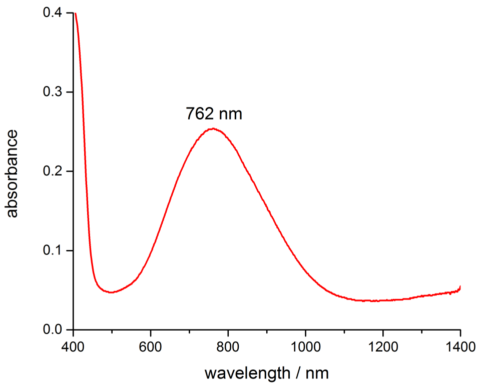

Figure S16. UV/vis/NIR spectrum of oxidised **TDPB** (0.2 mg/ml) doped with **AOT**, in ethanol

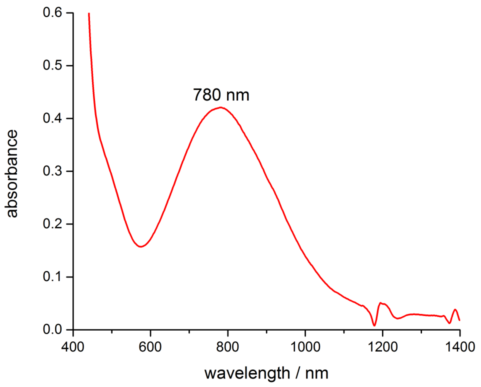

Figure S17. UV/vis/NIR spectrum of oxidised **TDPB** (0.3 mg/ml) doped with **BEHP**, in THF

6. Cyclic voltammetry

In acidic solution

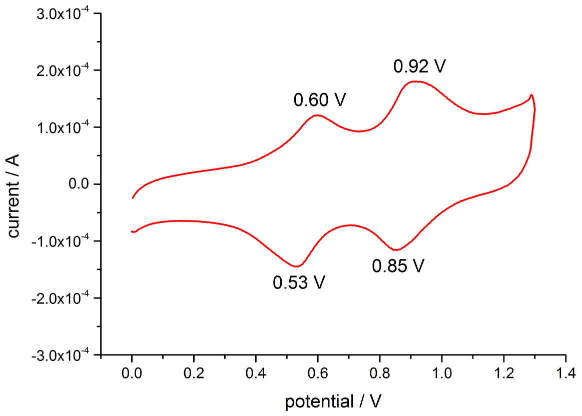

Figure S18. Cyclic voltammogram of **TDPB** in MeCN/$Bu_4N^+PF_6^-$ (0.1 M)/$HClO_4$ (0.01 M) at 293 K, scan rate 25 mV/s, potential vs. SCE calibrated against Fc/Fc^+ (0.43 V).

In neutral solution

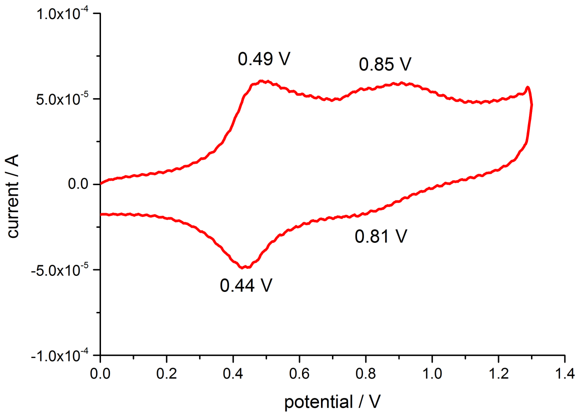

Figure S19. Cyclic voltammogram of **TDPB** in MeCN/$Bu_4N^+PF_6^-$ (0.1 M) at 293 K, scan rate 25 mV/s, potential vs. SCE calibrated against Fc/Fc^+ (0.43 V).

7. Density functional theory (DFT) modelling

Computational approach and methods

A molecular model of **TDPB** in the reduced state was constructed with initial bond angles and dihedral angles based on those found in crystal structures of oligo(aniline)s reported in the literature.[28] This initial geometry were optimised in *Gaussian*[31] using DFT with the B3LYP functional,[32] the 6-31G* basis set[33] and the built-in PCM solvation model.[34]

The optimised structure of reduced **TDPB** was then used as the starting point for the oxidised and doped states. Only *trans* isomers of quinonediimine groups were considered for oxidised species, as this is the geometry found in the solid state.[28] The structures of the three oxidised species are given in Scheme S2.

For the doped states, reduced **TDPB** was used without modification as the input structure, with the charge and spin set to the appropriate value for the doped species under consideration in each case. Although counterions are invariably associated with cationic doped oligo(aniline)s in solution and solid phases, in the absence of direct experimental evidence of the positions of these anions in relation to the oligomer, we modelled the doped states without anions.

We compared geometry optimisation by B3LYP and CAM-B3LYP and found the former performed better. In their initial report on the CAM-B3LYP functional, Handy and co-workers noted its superiority over B3LYP at modelling the excited states of molecules capable of significant charge-transfer (CT) excitations.[35] Tozer and co-workers confirmed the excellent performance of CAM-B3LYP at modelling CT excitations applies to a wide range of molecules.[36] For these reasons, we adopted a two-step approach in which the molecule undergoes geometry optimisation with B3LYP followed by an excited state calculation with CAM-B3LYP. UV-vis-NIR spectra were simulated using the built-in tool in *GaussView*.[34]

Reduced state

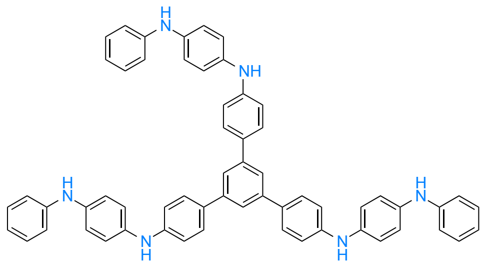

Scheme S3. Structure of the reduced state of **TDPB**

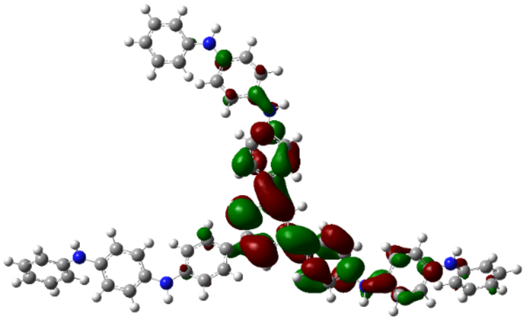

Figure S20. LUMO of reduced **TDPB**

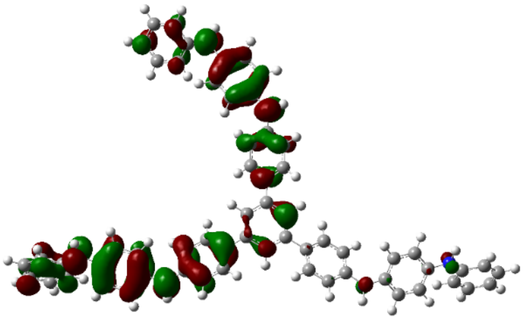

Figure S21. HOMO of reduced **TDPB**

Table S2. DFT-calculated frontier molecular orbitals of the reduced state of **TDPB**

MO number	MO label	MO energy / au
227	LUMO+1	−0.03114
226	LUMO	−0.03146
225	HOMO	−0.17252
224	HOMO−1	−0.17314
223	HOMO−2	−0.17432

Table S3. TD-DFT calculated electronic transitions of the reduced state of **TDPB**

Excited state	ΔE / eV	λ / nm	f	Major contributions
1	4.00	310	1.52	HOMO → LUMO+2 (21%)<br>HOMO−2 → LUMO+1 (20%)
2	4.01	309	3.06	HOMO−2 → LUMO (24%)<br>HOMO−1 → LUMO+2 (22%)
3	4.16	298	0.33	HOMO−1 → LUMO (27%)<br>HOMO → LUMO (25%)<br>HOMO−2 → LUMO+2 (22%)

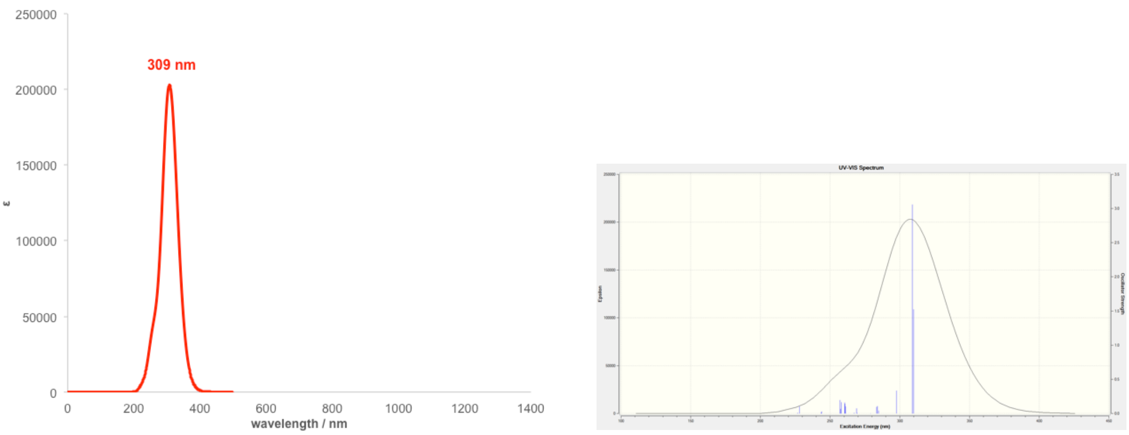

Figure S22. Simulated UV/vis/NIR spectrum of reduced **TDPB** based on the TD-DFT calculation

Oxidised state 1

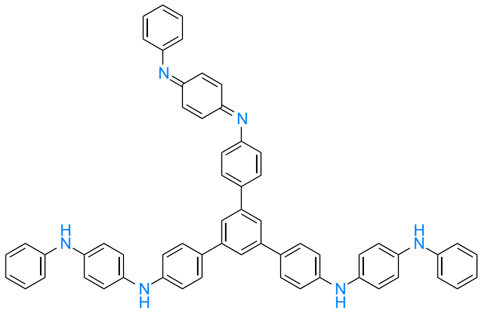

Scheme S4. Structure of the first oxidised state of **TDPB**

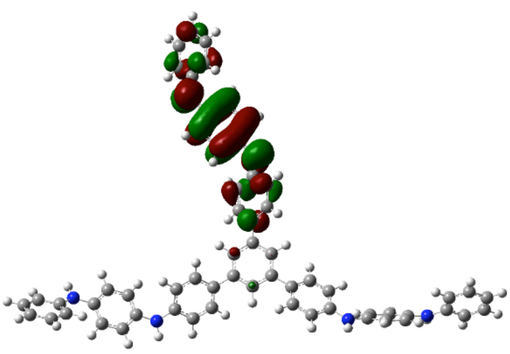

Figure S23. LUMO of **TDPB** with one arm oxidised

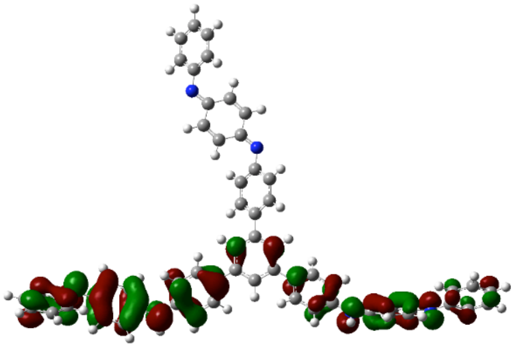

Figure S24. HOMO of **TDPB** with one arm oxidised

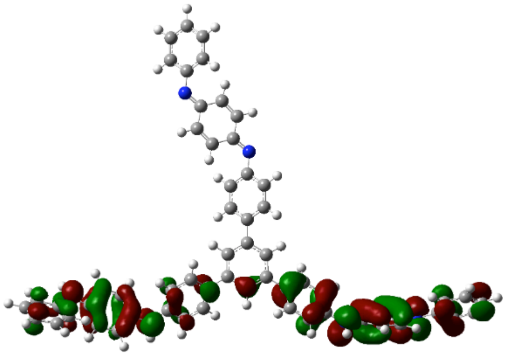

Figure S25. HOMO−1 of **TDPB** with one arm oxidised

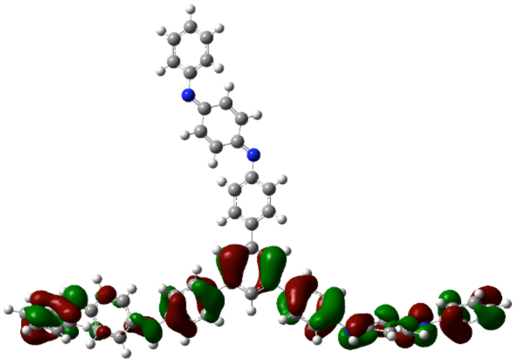

Figure S26. HOMO−2 of **TDPB** with one arm oxidised

Table S4. DFT-calculated frontier molecular orbitals of **TDPB** with one arm oxidised

MO number	MO label	MO energy / au
226	LUMO+1	−0.03319
225	LUMO	−0.10749
224	HOMO	−0.17401
223	HOMO−1	−0.17459
222	HOMO−2	−0.20432

Table S5. TD-DFT calculated electronic transitions of the reduced state of **TDPB** with one arm oxidised

Excited state	ΔE / eV	λ / nm	f	Major contributions
1	2.79	445	1.04	HOMO−2 → LUMO (61%)<br>HOMO−4 → LUMO (24%)
5	4.02	308	1.08	HOMO−17 → LUMO (26%)<br>HOMO−16 → LUMO (14%)
6	4.03	308	3.27	HOMO−1 → LUMO+2 (31%)<br>HOMO → LUMO+3 (15%)

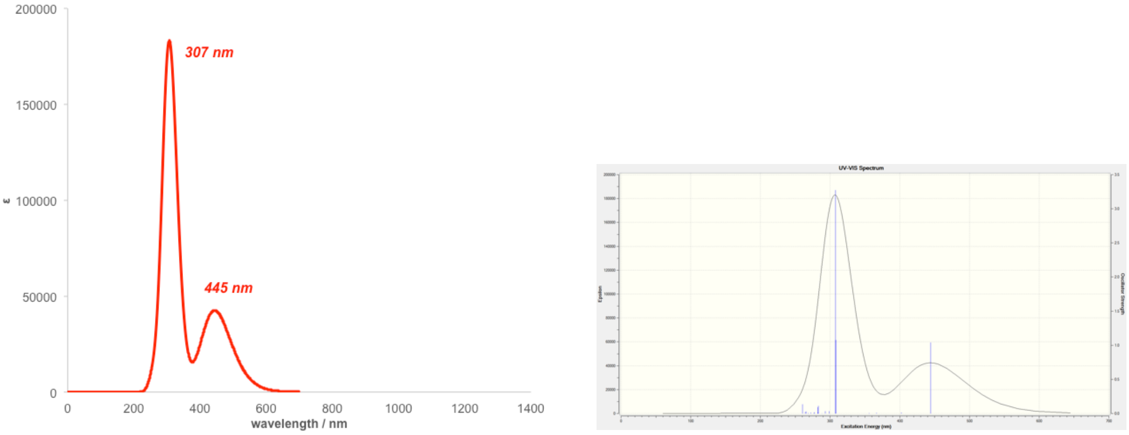

Figure S27. Simulated UV/vis/NIR spectrum of **TDPB** with one arm oxidised, based on the TD-DFT calculation

Oxidised state 2

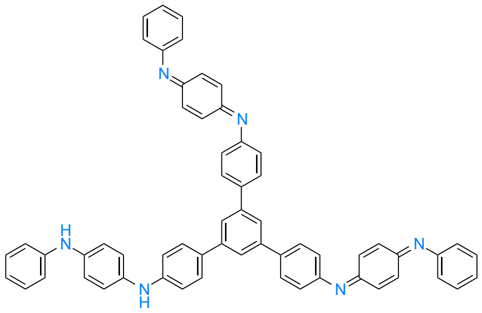

Scheme S5. Structure of the second oxidised state of **TDPB**

Table S6. DFT-calculated frontier molecular orbitals of **TDPB** with two arms oxidised

MO number	MO label	MO energy / au
225	LUMO+1	−0.10769
224	LUMO	−0.17420
223	HOMO	−0.20534
222	HOMO−1	−0.20714
221	HOMO−2	−0.21242

Table S7. TD-DFT calculated electronic transitions of **TDPB** with two arms oxidised

Excited state	Δ*E* / eV	*λ* / nm	*f*	Major contributions
1	2.75	450	1.43	HOMO−1 → LUMO+1 (45%)<br>HOMO−3 → LUMO (19%)<br>HOMO−2 → LUMO (16%)
2	2.82	440	0.68	HOMO−1 → LUMO (41%)<br>HOMO−3 → LUMO+1 (19%)<br>HOMO−2 → LUMO+1 (15%)
7	3.98	311	1.85	HOMO−15 → LUMO+1 (16%)<br>HOMO−16 → LUMO+1 (14%)<br>HOMO → LUMO+3 (12%)
8	4.01	309	1.86	HOMO → LUMO+3 (14%)<br>HOMO−15 → LUMO (13%)<br>HOMO−16 → LUMO+1 (11%)

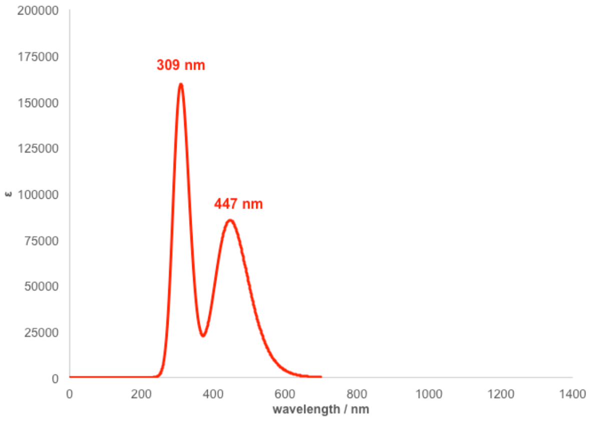

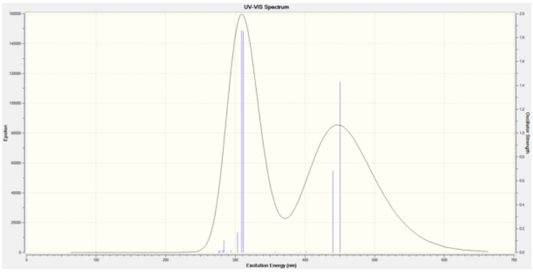

Figure S28. Simulated UV/vis/NIR spectrum of **TDPB** with two arms oxidised, based on the TD-DFT calculation

Oxidised state 3

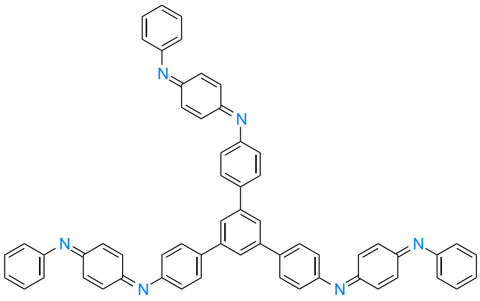

Scheme S6. Structure of the third oxidised state of **TDPB**

Table S8. DFT-calculated frontier molecular orbitals of **TDPB** with three arms oxidised

MO number	MO label	MO energy / au
224	LUMO+1	−0.10793
223	LUMO	−0.10802
222	HOMO	−0.20824
221	HOMO−1	−0.20835
220	HOMO−2	−0.21312

Table S9. TD-DFT calculated electronic transitions of **TDPB** with three arms oxidised

Excited state	Δ*E* / eV	*λ* / nm	*f*	Major contributions
1	2.75	450	1.32	HOMO−2 → LUMO (22%)<br>HOMO−4 → HOMO (17%)
2	2.78	446	1.74	HOMO−3 → LUMO (23%)<br>HOMO−4 → HOMO−1 (18%)
7	3.99	311	1.38	HOMO−16 → HOMO (14%)<br>HOMO−18 → LUMO (13%)
8	4.03	308	1.77	HOMO−17 → LUMO (16%)<br>HOMO−2 → HOMO−1 (11%)

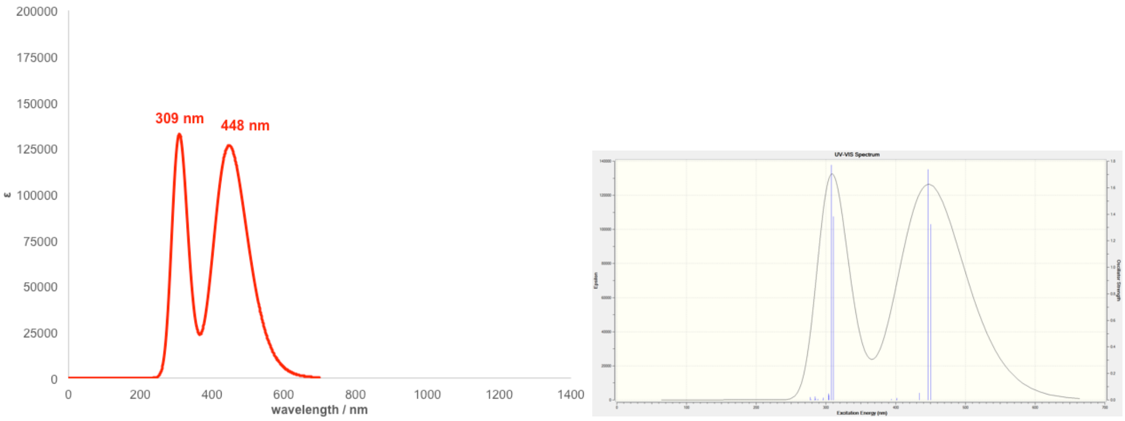

Figure S29. Simulated UV/vis/NIR spectrum of **TDPB** with three arms oxidised, based on the TD-DFT calculation

Doped states

Radical cation

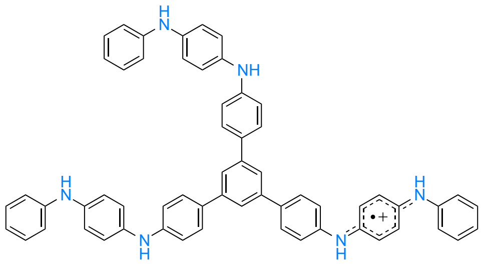

Scheme S7. Structure of the radical cation of **TDPB**

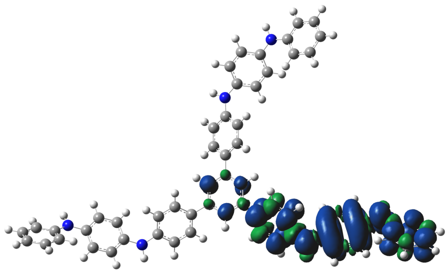

Figure S30. Spin density of the radical cation of **TDPB**

Table S10. TD-DFT calculated electronic transitions of the radical cation of **TDPB**

Excited state	Δ*E* / eV	*λ* / nm	*f*	Major contributions
1	1.61	772	0.74	HOMO−4 (β) → LUMO (β)
14	3.32	374	0.14	HOMO−4 (α) → LUMO (α)<br>HOMO−15 (β) → LUMO (β)
20	3.65	340	0.25	HOMO−4 (α) → LUMO (α)<br>HOMO−18 (β) → LUMO (β)

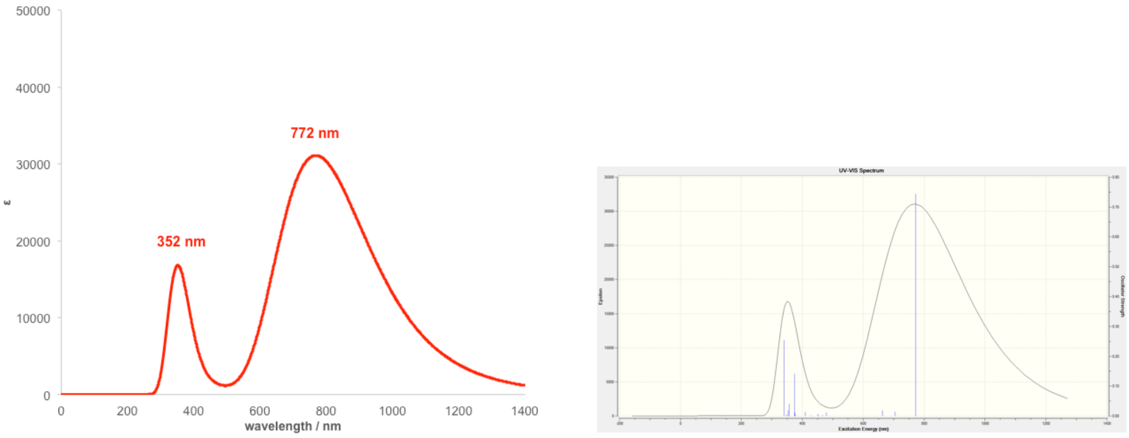

Figure S31. Simulated UV/vis/NIR spectrum of the radical cation of **TDPB** based on the TD-DFT calculation

Singlet dication

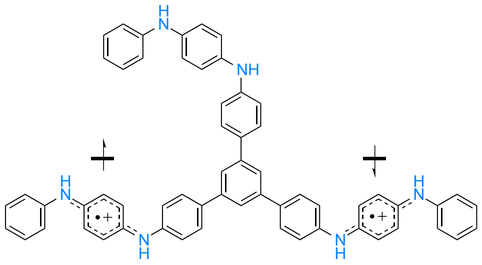

Scheme S8. Structure of the singlet dication of **TDPB**

Table S11. TD-DFT calculated electronic transitions of the singlet dication of **TDPB**

Excited state	Δ*E* / eV	*λ* / nm	*f*	Major contributions
3	1.24	1002	0.65	HOMO−3 → LUMO (49%)<br>HOMO−2 → LUMO (39%)
4	1.32	941	0.53	HOMO−2 → LUMO (51%)<br>HOMO−3 → LUMO (45%)
5	1.55	801	0.49	HOMO−4 → LUMO (91%)
20	3.75	330	1.44	HOMO−1 → LUMO+1 (42%)<br>HOMO → LUMO+2 (35%)

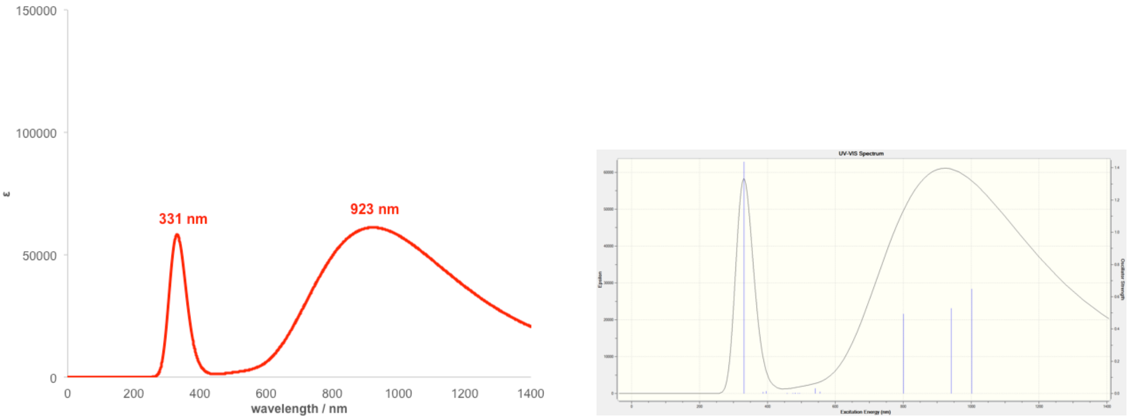

Figure S32. Simulated UV/vis/NIR spectrum of the singlet dication of **TDPB** based on the TD-DFT calculation

Triplet dication

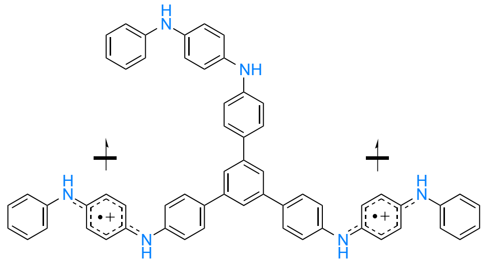

Scheme S9. Structure of the triplet dication of **TDPB**

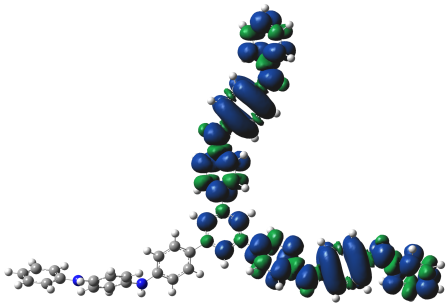

Figure S33. Spin density of the triplet dication of **TDPB**

Table S12. TD-DFT calculated electronic transitions of the triplet dication of **TDPB**

**Excited state**	**ΔE / eV**	**λ / nm**	***f***	**Major contributions**
1	1.60	773	0.57	HOMO−2 (β) → LUMO (β)<br>HOMO−3 (β) → LUMO (β)<br>HOMO−2 (β) → LUMO+1 (β)<br>HOMO−3 (β) → LUMO+1 (β)
2	1.66	745	0.98	HOMO−2 (β) → LUMO (β)<br>HOMO−3 (β) → LUMO (β)<br>HOMO−2 (β) → LUMO+1 (β)<br>HOMO−3 (β) → LUMO+1 (β)
17	3.27	380	0.12	HOMO−2 (α) → LUMO (α)
20	3.37	368	0.19	HOMO−2 (α) → LUMO+1 (α)<br>HOMO−3 (α) → LUMO (α)

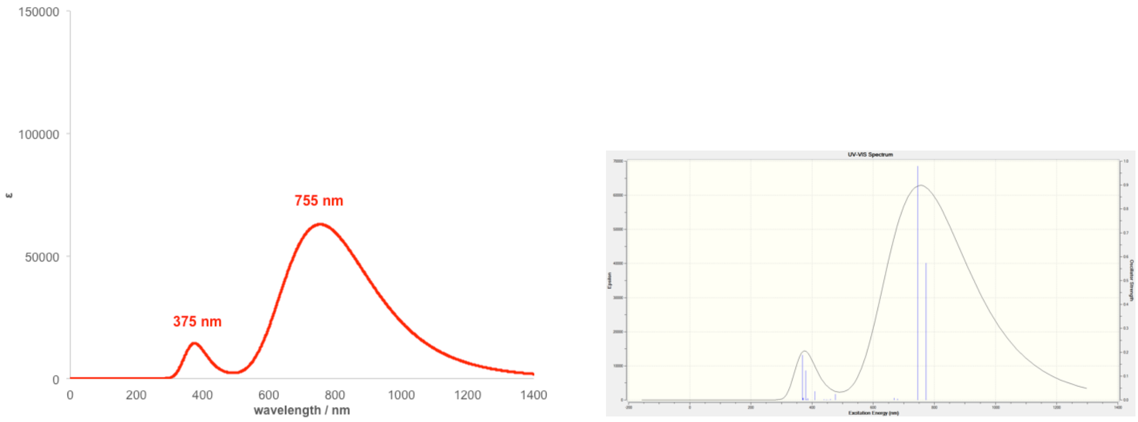

Figure S34. Simulated UV/vis/NIR spectrum of the triplet dication of **TDPB** based on the TD-DFT calculation

Doublet trication

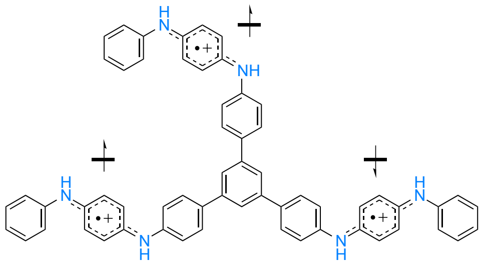

Scheme S10. Structure of the doublet trication of **TDPB**

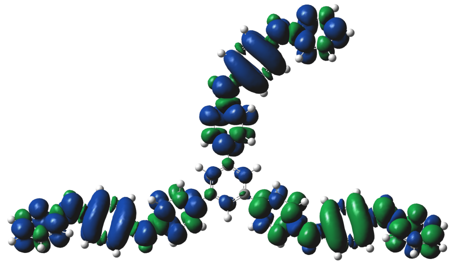

Figure S35. Spin density of the doublet trication of **TDPB**

Table S13. TD-DFT calculated electronic transitions of the doublet trication of **TDPB**

**Excited state**	**Δ*E* / eV**	***λ* / nm**	***f***	**Major contributions**
1	1.62	766	0.44	HOMO−1 (β) → LUMO (β)<br>HOMO−2 (β) → LUMO (β)<br>HOMO−2 (α) → LUMO (α)
2	1.66	748	1.69	HOMO−2 (α) → LUMO (α)<br>HOMO−2 (β) → LUMO+1 (β)<br>HOMO−1 (β) → LUMO+1 (β)
12	3.03	409	0.13	HOMO (β) → LUMO+1 (β)<br>HOMO−5 (α) → LUMO (α)
19	3.35	370	0.27	HOMO−1 (β) → LUMO+1 (β)<br>HOMO−2 (β) → LUMO+1 (β)

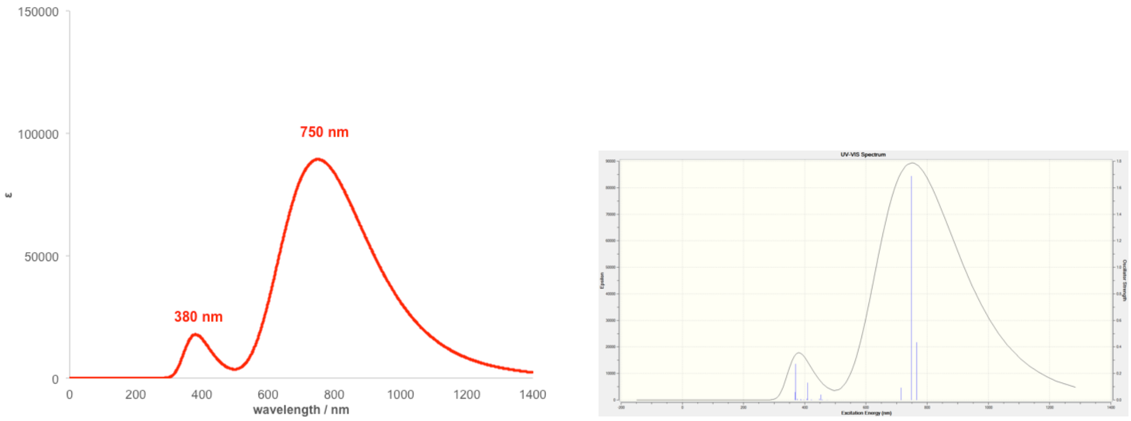

Figure S36. Simulated UV/vis/NIR spectrum of the doublet trication of **TDPB** based on the TD-DFT calculation

Quartet trication

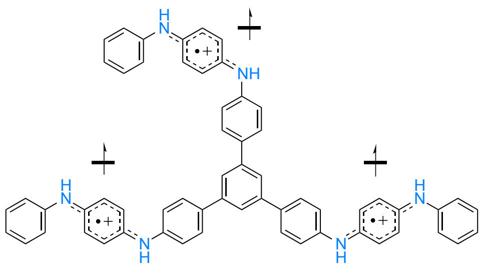

Scheme S11. Structure of the quartet trication of **TDPB**

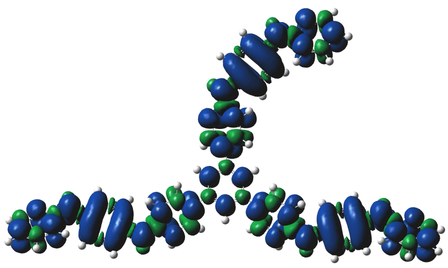

Figure S37. Spin density of the quartet trication of **TDPB**

Table S14. TD-DFT calculated electronic transitions of the quartet trication of **TDPB**

Excited state	Δ*E* / eV	*λ* / nm	*f*	Major contributions
1	1.62	766	0.51	HOMO (β) → LUMO (β)<br>HOMO−2 (β) → LUMO (β)<br>HOMO−1 (β) → LUMO (β)
2	1.66	748	1.65	HOMO−1 (β) → LUMO+2 (β)<br>HOMO (β) → LUMO+1 (β)<br>HOMO−2 (β) → LUMO+2 (β)
17	3.35	370	0.51	HOMO−2 (α) → LUMO+1 (α)
18	3.35	370	0.17	HOMO−2 (α) → LUMO (α)<br>HOMO−13 (α) → LUMO+8 (α)

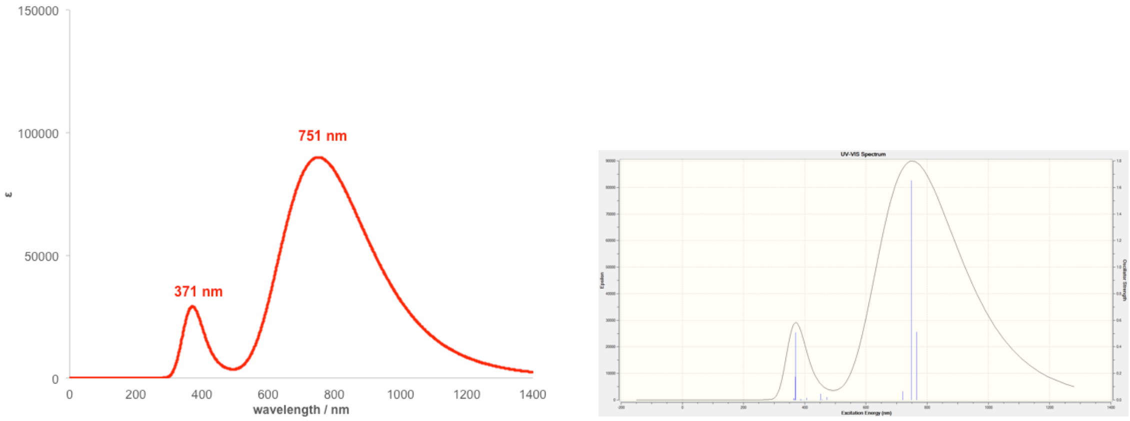

Figure S38. Simulated UV/vis/NIR spectrum of the quartet trication of **TDPB** based on the TD-DFT calculation

8. Simulated and experimental spectra of a linear analogue, DPPD

Reduced state

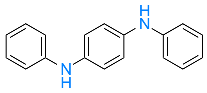

Scheme S12. Structure of the reduced state of **DPPD**

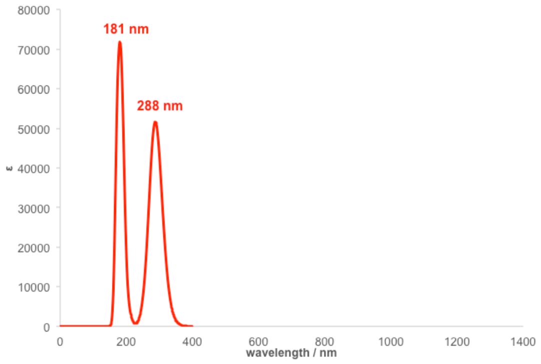

Figure S39. TD-DFT-simulated UV/vis/NIR spectrum of reduced **DPPD**

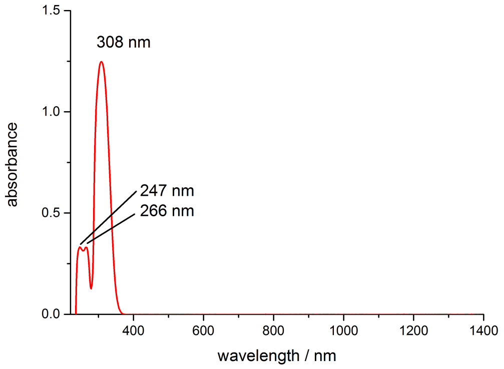

Figure S40. Experimental UV/vis/NIR spectrum of reduced **DPPD** in THF

Oxidised state

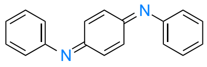

Scheme S13. Structure of the reduced state of **DPPD**

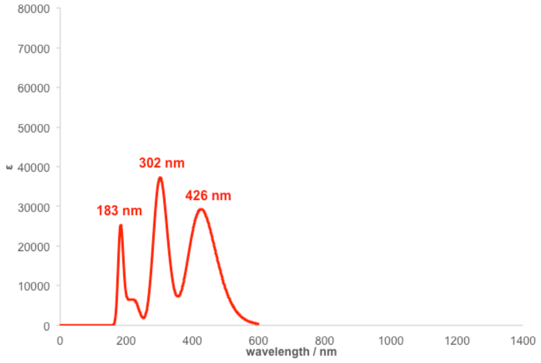

Figure S41. TD-DFT-simulated UV/vis/NIR spectrum of oxidised **DPPD**

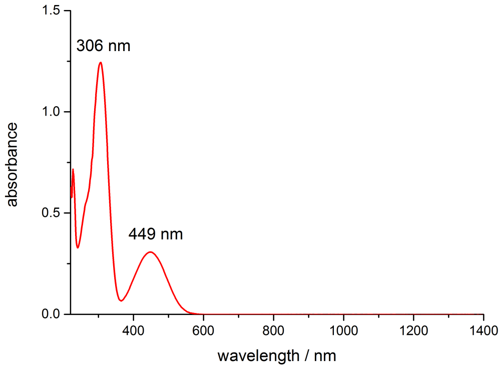

Figure S42. Experimental UV/vis/NIR spectrum of oxidised **DPPD** in THF

Radical cation

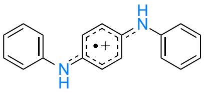

Scheme S14. Structure of the radical cation of **DPPD**

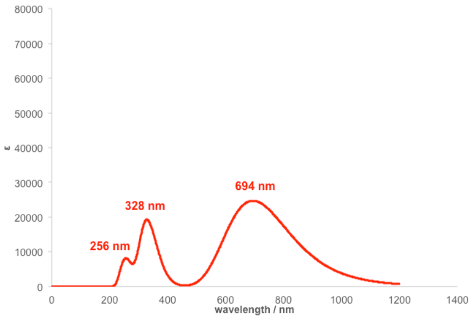

Figure S43. TD-DFT-simulated UV/vis/NIR spectrum of the radical cation of **DPPD**

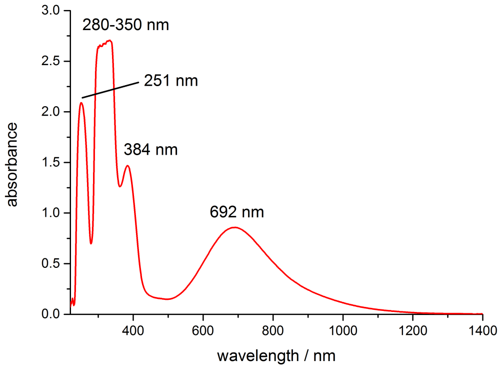

Figure S44. Experimental UV/vis/NIR spectrum of the radical cation of **DPPD** in THF

Summary

Table S15. Comparison of experimental and TD-DFT-simulated maxima for **DPPD** and **TDPB**

	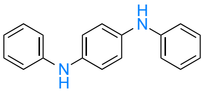 **DPPD**		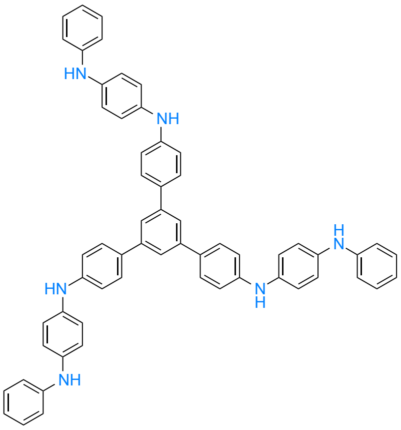 **TDPB**	
**State**	**Simulated maximum / nm**	**Experimental maximum / nm**	**Simulated maximum / nm**	**Experimental maximum / nm**
Reduced	288	308	309	342
Oxidised	426	449	445	469
Radical cation	694	692	772	778
